# Old Friends with Unexploited Perspectives: Current Advances in Mesenchymal Stem Cell-Based Therapies in Asthma

**DOI:** 10.1007/s12015-021-10137-7

**Published:** 2021-03-01

**Authors:** Marlena Tynecka, Marcin Moniuszko, Andrzej Eljaszewicz

**Affiliations:** 1 Department of Regenerative Medicine and Immune Regulation, Medical University of Bialystok, ul. Waszyngtona 13, 15-269 Białystok, Poland; 2 Department of Allergology and Internal Medicine, Medical University of Bialystok, ul. M. Skłodowskiej-Curie 24A, Białystok, 15-276 Poland

**Keywords:** Mesenchymal stem cells, Asthma, Immune regulation, Experimental asthma, Stem cells

## Abstract

Mesenchymal stem cells (MSCs) have a great regenerative and immunomodulatory potential that was successfully tested in numerous pre-clinical and clinical studies of various degenerative, hematological and inflammatory disorders. Over the last few decades, substantial immunoregulatory effects of MSC treatment were widely observed in different experimental models of asthma. Therefore, it is tempting to speculate that stem cell-based treatment could become an attractive means to better suppress asthmatic airway inflammation, especially in subjects resistant to currently available anti-inflammatory therapies. In this review, we discuss mechanisms accounting for potent immunosuppressive properties of MSCs and the rationale for their use in asthma. We describe in detail an intriguing interplay between MSCs and other crucial players in the immune system as well as lung microenvironment. Finally, we reveal the potential of MSCs in maintaining airway epithelial integrity and alleviating lung remodeling.

**Graphical abstract:**

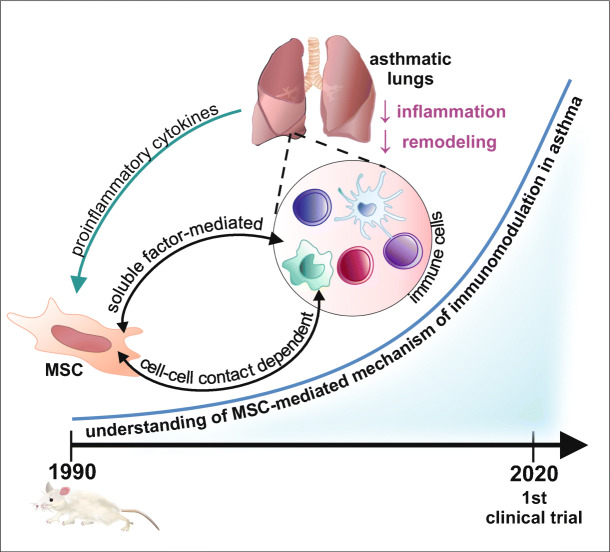

## Introduction

Significant progress in our understanding of stem cell biology accompanied by development of technologies enabling the induction of pluripotency in somatic cells opened new ways to develop stem cell-based therapies for currently incurable diseases [1–5]. However, initial optimism was quickly disturbed by ethical dilemmas and significant safety issues. Apart from crucial ethical dilemmas concerning embryonic stem cells, the most controversial issues inhibiting the wider use of adult stem cells and induced pluripotent stem cells (iPCs) relate to their stability and long-term effects of their application [6–13]. In fact, to date, only two stem cell-based therapies were approved by FDA (U.S. Food and Drug Administration) and EMA (European Medicines Agency). These are hematopoietic stem cell transplantation, available at: https://www.fda.gov/vaccines-blood-biologics/cellular-gene-therapy-products/approved-cellular-and-gene-therapy-products [14] and limbal stem cell therapy used for for corneal transplant vision recovery, available at: https://www.ema.europa.eu/en/news/first-stem-cell-therapy-recommended-approval-eu [15]. Other stem cell-based therapies are still tested in clinical trials or applied as medical experiments [16, 17]. Therefore, they still represent patients and medical professionals’ unfulfilled dreams rather than a widely available therapeutic option.

This holds true for patients suffering from advanced inflammatory diseases [18–21], poorly healing injuries [22–24], and irreversible tissue/organ damage [25–28], including patients with advanced suboptimally controlled or even uncontrolled respiratory diseases such as acute respiratory disstres syndrome (ARDS) [29], idiopathic pulmonary fibrosis [30] and severe persistent asthma with lung remodeling [31–36].

Inhaled corticosteroids (ICS) are the mainstay in asthma therapy as they effectively control symptoms and prevent exacerbations in the majority of patients. Asthmatic airway inflammation can be further alleviated by allergen-specific immunotherapy in allergic asthmatics and biological therapy in patients with severe eosinophil asthma [37–39]. In contrast, neutrophilic steroid-resistant asthma represents a significant therapeutic challenge. Notably, poorly controlled and uncontrolled asthmatic patients are consuming over half of the healthcare resources planned for asthma management in highly developed countries [40–42]. Therefore, there is still a substantial need for novel effective therapeutic options that may help better manage poorly responding and non-responding asthmatics. Thus, stem cell-based therapies, including mesenchymal stem cells (MSC) and iPCs, have been proposed as potential therapeutic options in severe asthma. In fact, anti-inflammatory effects of MSCs have been observed and described over 30 years ago [43, 44]; however, our understanding of the mechanisms of their beneficial effects in respiratory diseases remained elusive, making MSCs old good friends with still unexploited potential.

In this review, we summarized the current understanding of the mechanisms of MSC-mediated regulation of inflammatory processes with particular focus on the advances in their beneficial effects in asthmatic lung inflammation.

## Mesenchymal Stem Cells

The mesenchymal stem cells (MSCs), also reffered to us as “mesenchymal stromal cells” and “medicinal signaling cells” are multipotent stromal cells [45]. They have been identified and isolated from various human tissues, including adipose tissue [46], bone marrow [47, 48], Wharton’s jelly [49], cord blood [50, 51], amniotic fluid [52, 53], amniotic membrane [54], dental pulp [55], endometrium [56, 57], peripheral blood [58, 59], salivary gland [60], and synovial fluid [61]. Although MSCs harvested from different tissues may slightly differ in their phenotype and functional properties, the minimal criteria for their definition have been proposed by the International Society for Cellular Therapy. According to the consensus, MSCs should: i) be positive for CD29, CD71, CD73, CD90, CD105, CD271 and lack of CD14, CD34, CD45, and human leukocyte antigen-DR isotope (HLA-DR) expression; ii) exhibit plastic adherence; and iii) possess the ability to differentiate in vitro into mesodermal lineage cells, including osteoblasts, chondrocytes, and adipocytes [62, 63]. Despite, substantial morphological and functional similarity among various subsets of MSCs, their differentiation capability, proliferation efficacy, immunomodulatory and regenerative properties may differ depending on their tissue sources. Nowadays, mesenchymal stem cells harvested from adipose tissue, bone marrow, umbilical cord blood, and Wharton’s jelly represent the most extensively described MSCs subpopulations. However, to date, the reports comparing functional properties of MSCs from different sources in the same laboratory conditions are rare [64]. According to available resources, adipose tissue-derived MSCs possess similar or even more potent immunomodulatory properties compared to bone marrow-derived MSCs [65–67]. In contrast, umbilical cord blood-derived and Wharton jelly’s derived MSCs show higher proliferation efficacy than MSCs isolated from bone marrow and adipose tissue [68, 69]. Thus, slightly different functional characteristics of MSCs derived from particular tissues opened scientific debate considering better and worse “candidates” to implement in cell-based therapies.

Interestingly, recently single-cell transcriptomic profiling of the lung allowed to confirm previously reported presence of lung resident mesenchymal stem cells (LR-MSCs) [70, 71]. Although phenotypically similar to bone marrow-derived MSCs, LR-MSCs possess distinct transcriptomic profiles, which may indicate their functional diversity and resulting from the local microenvironmental stimulus. Unfortunately, to date, our understanding of the role of LR-MSCs is minimal. It seems, however, that they can play both either beneficial or pathological roles in lung inflammation dependent on the dynamic changes occurring within local microenvironemnet. In fact, LR-MSCs represent an important source of growth factors such as keratinocyte growth factor (KGF) [72], fibroblast growth factor-10 (FGF-10) [73], and hepatocyte growth factor (HGF) [74, 75], crucial for the preservation of lung homeostasis. In several pathological conditions, LR-MSCs may serve as regulators of lung inflammation, inhibiting Th17 immune responses and increase T regulatory cell (Treg) activity [72]. On the other hand, transforming growth factor-beta (TGFβ)-triggered activation of the Wnt/β-catenin signaling may lead to differentiation of LR-MSCs towards myofibroblasts. This would indicate to LR-MSCs as cells which after having received some specific signals could contribute to the process of lung remodeling [76]. To date, however, it remains elusive whether this putative profibrotic potential of LR-MSCs can truly play an actual role in asthma pathogenesis [76–78]. Further understanding of the role of LR-MSCs in airway inflammation and remodeling is still warranted as it can open new ways for better asthma management.

Introduction of biotechnological methods, allowing to induce pluripotency by genetic reprogramming of somatic cells and their further maturation towards multipotent stem cells allowed to establish iPSC-derived MSC (iPSC-MSC). Notably, they can be produced by non-integrating episomal methods and acquire the capacity to reproduce without losing their vital functional properties [79, 80]. Therefore, iPSC-MSCs lack the reported weaknesses of natural (tissue-derived) MSCs, namely limited proliferative potential, standardization difficulty, loss of differentiation capacity in the late passage, decrease in therapeutic efficacy during expansion [81, 82]. On the other hand, induction of iPSC-MSC raises concerns about their further stability in clinical settings, e.g. after transplantation. Unfortunately, genetic modifications employed at the iPSC level may lead to oncogene activation resulting in genetic and epigenetic abnormalities and, in consequence, leading to tumorigenesis [83]. Despite these concerns, iPSC-MSCs hold high therapeutic potential, which has been demonstrated in some preclinical studies [83]. It provides cautious hope for the future application of iPSC-MSCs in clinical settings following careful addressing safety concerns. To date, only two studies on iPSC-MSC-based treatment have been registered in the clinicaltrials.gov database. Mesenchymoangioblast- derived mesenchymal stem cells were tested in steroid-resistant Graft versus Host Disease (GvHD) [84], and acute distress respiratory syndrome in the course of COVID-19 [85].

In fact, according to available preclinical data, iPSC-MSCs reduce both Th2-driven and non-Th2-driven airway inflammation. Similarly to natural MSCs, administration of iPSC-MSCs decreased mucus production and immune cells’ infiltration within lungs as well as interleukin (IL)-4, IL-5, and IL-13 levels in bronchoalveolar lavage fluid (BALF). On the other hand, the limitation of non-Th2-driven inflammation was associated with a significant decrease in Th17 cell infiltration. Importantly, iPSC-MSCs have also been shown to reduce epithelium-derived alarmins, namely IL-33 and thymic stromal lymphopoietin (TSLP) [86–88].

iPSC-MSC represents a significant development of the research on the immunosuppressive activities of MSCs. Nevertheless, iPSC-MSC’s application for the regulation of asthmatic inflammation requires further progress in understanding their long-term stability and function.

## MSC-Mediated Modulation of Immune Responses

The MSCs may exert diverse immunomodulatory effects upon administration into different inflammatory conditions [89]. MSCs-mediated immune regulation seems to depend strictly on the local microenvironment, usually associated with the disease stage [90, 91]. The numerous reports have presented the MSCs-mediated immunoregulatory effects associated with: i) reduction of CD4+ and CD8+ T cell proliferation, [92] ii) inhibition of monocyte and CD34+ cells maturation towards proinflammatory macrophages and dendritic cells (DCs) [93], iii) recruitment Tregs and induction of effector T cell functional plasticity [94, 95], iv) inhibition of cytotoxicity and proliferation of natural killer (NK) cells [96, 97], and v) limitation of B cells maturation and antibody production [98, 99].

Despite an accumulating body of evidence showing the effectiveness of MSCs administration in preclinical and clinical settings, several studies reported failures in their application for immune response modulation in Graft-versus-Host Disease (GVHD) [100], Crohn Disease [101], and Systemic Lupus Erythematosus (SLE) [102]. However, it seems that the lack of desired immunosuppressive effects may be caused by inadequate MSCs “licensing” by the inflammatory mediators and/or untimely cell administration [89, 90]. More specifically, a proper “licensing” (also known in the term “priming” or “preconditioning”) is described as a process to prepare the cells to acquire specific properties in response to particular microenvironment components and conditions. To date, several reports demonstrated that hypoxia [103], TLRs agonists [104, 105], and proinflammatory mediators, including cytokines as crucial stimuli to enhance MSCs’ immunomodulatory and regenerative properties [106–108]. In fact, the presence of cytokines characteristic for non-type two inflammation, interferon-gamma (IFNγ), IL-1α, and IL-1β [20, 94], induces MSC immunosuppressive functions. To date, it remains elusive whether type-2 related cytokines such as IL-4, IL-5, and IL-13 may directly induce regulatory properties of MSCs; however, their effectiveness in limiting Th2-driven inflammation is well established. Under resting conditions, MSCs have been shown to exert antiapoptotic effects and may act as functional “supporters” of various immune cells, such as T cells, B cells, and plasma cells [109]. Interestingly, the MSC polarization towards the proinflammatory phenotype has been observed in cells stimulated with Toll-like receptor (TLRs) agonists, such as a prototypic TLR2 ligand Pam3Cys [104, 110]. Thus, it seems that the immunosuppressive properties of MSCs are induced mainly by the proinflammatory cytokines rather than the constitutive function of these cells [109].

**Fig. 1:**
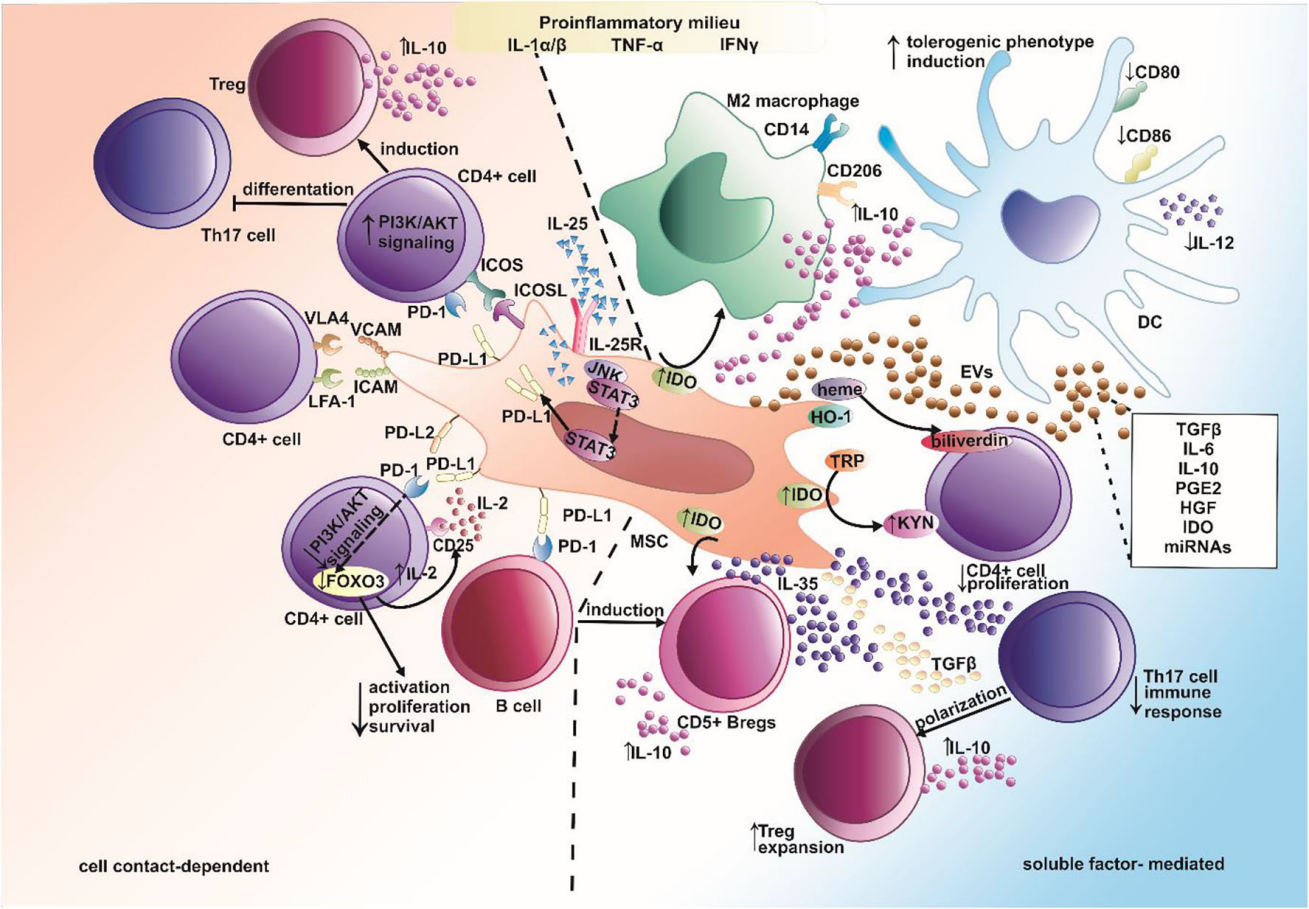
The direct and indirect immunomodulatory mechanism of mesenchymal stem cells. Mesenchymal stem cells exert the immunosuppressive effects by direct (cell-to-cell dependent; marked on the orange background) and indirect (secretome-mediated; marked on the blue background) interactions with immune cells. Induction of immunosuppressive properties of MSCs requires proinflammatory signaling mediated by IL-1α/β, TNF-α, IFNγ, among others. Breg- B regulatory cell; DC- dendritic cell; EVs- extracellular vesicles; FOXO3- forkhead box O3; HGF- hepatocyte growth factor; HO-1-heme oxygenase 1; ICAM- intracellular adhesion molecule 1; ICOS- inducible costimulator; ICOSL- idnucible costimulator ligand; IDO-indoleamine 2,3-dioxygenase; IFNγ- interferon gamma; IL-(1α, 1β, 2, 6, 10, 12, 25, 35)- interleukin 1α, 1β, 2, 6, 10, 12, 25, 35; IL-25R- interleukin 25 receptor; JNK- c-Jun N-terminal kinase; KYN-kynurenine; LFA-1- lymphocyte function-associated antigen 1; MSC-mesenchymal stem cell; PD-1- programmed cell death receptor 1; PD-L(1/2)- programmed death-ligand 1/2; PGE2- prostaglandin E2; PI3K/ AKT- phosphoinositide 3-kinase/ protein kinase B; STAT3- signal transducer and activator of transcription 3; TGFβ- transforming growth factor β; TNF-α- tumor necrosis factor α; Treg- T regulatory cell; TRP- tryptophan; VCAM1- vascular cell adhesion protein 1; VLA4- very late antigen 4, ←activation/ induction; ├ inhibition; ↑increase; ↓decrease

Notably, MSC-mediated interactions leading to the regulation of inflammatory responses are associated with both direct (cell-to-cell dependent) and indirect (soluble factor release dependent) mechanisms (Fig. 1) [111].

## Cell Contact-Dependent Immune-Modulatory Mechanisms

The processes underlying cell-dependent mechanisms of MSCs-mediated immunosuppression involve a number of immunomodulatory membrane-bound proteins, including costimulatory receptors, membrane-bound cytokines, and small molecules [111–113]. This part will summarize the current understanding of the direct mutual interactions between MSCs and different immune cells.

Attenuation of T cell proliferation and induction of Tregs has been recognized as one of the hallmarks of MSCs immunomodulatory properties. This effect seems to be controlled by INFγ [94]. In fact, IFNγ signaling in MSCs was shown to induce higher expression of checkpoint proteins, namely programmed cell death-ligand 1 (PD-L1, also known as B7-H1) and programmed cell death-ligand 2 (PD-L2, B7-DC) [95, 114, 115]. Both ligands interact with PD-1 on T and B cells and inhibit their T- cell receptor (TCR) and B cell receptor (BCR)-mediated activation, respectively [116].

In T cells, MSC dependent PD-1 signaling induces reduction of TCR-mediated IL-2 production and, in consequence, decrease their proliferation and CD4 + CD25+ cell survival. In addition, PD-L1/PD-1 interaction was shown to downregulate Th17 cell activity and to reduce naïve CD4+ T cell differentiation towards Th1 and Th17 cells. The effects of Th17 cell-mediated suppression seems to be regulated by the IL-25/STAT3/PD-L1 axis [117]. In the in vivo settings, a decrease of proinflammatory (Th1/Th17) T cell infiltrate, after MSC administration, is usually associated with an increase in regulatory T cells’ frequency. Recently, MSC mediated PD-1/PD-L1 and ICOS-ICOSL interactions were proposed to play a central role in the induction of conventional T cell plasticity and induction of regulatory like phenotype. MSC induced regulatory like T cells, possess stable immune-suppressive properties, and displayed DNA methylation profiles resembling natural Tregs [118, 119]. It seems that this functional conversion requires direct cell-to-cell interaction. Moreover, MSCs adhesion was shown to be crucial to induce immunosuppressive effects of MSC derived soluble factors, including lipid mediators such as prostaglandin E2 (PGE2), nitric oxide (NO), cytokines (IL-2, IL-10, TSG-6), and indoleamine 2,3-dioxygenase (IDO); (for more details, please see Soluble Factor Mediated Immunomodulation section) [95, 113, 120–122]. VCAM-1 (vascular cell adhesion molecule 1) and ICAM-1 (intracellular adhesion molecule 1) seem to play an essential role in this process. Both receptors are constitutively expressed on MSCs, and their expression rises under IFNγ, tumor necrosis factor alpha (TNF-α), IL-1α, and IL-1β, stimulation [123].

In contrast to T cells, to date, the mechanisms of contact-dependent MSC-mediated regulation of B cell responses remain elusive. In fact, the MSC-mediated effect on B cells, similarly to antigen-presenting cells (APC), namely monocytes and dendritic cells, has often been attributed to MSC-released mediators and will be discussed in the following section. However, some reports showed that MSC reduces plasma blasts formation and promotes immune suppressive and tolerogenic regulatory B cell (Breg) differentiation [98, 124, 125]. Interestingly, the latter effect seems to be controlled by direct cellular interaction but after T cell-mediated proinflammatory cytokine conditioning of MSC [125, 126].

## Soluble Factor-Mediated Immunomodulation

Besides the importance of contact-dependent effects, the vast majority of to date published reports describe MSC-mediated indirect immunosuppressive mechanisms. MSC-derived secretome consists of proteins, small molecules, and extracellular vesicles (EVs), released into extracellular space, acting as orchestrator of immune responses. Here, we summarize current advances in the understanding of the mechanisms of paracrine-mediated immune-modulation [127].

Similarly to previously described cell-dependent mechanisms, MSC licensing by inflammatory cytokines is required to activate their immune-modulatory factors’ secretion. In the presence of IFNγ, MSCs release high amounts of IDO, which metabolizes the degradation of tryptophan to toxic catabolites accumulation, namely kynurenine, L-tryptophan, kynurenic acid, quinolinic acid, and anthranilic acid [122]. However, only the kynurenine impairs the effector function and proliferation of T cells [122]. Notably, the IDO-mediated effect on tryptophan depletion has been identified only locally [89, 127]. Therefore, it seems that cell-to-cell adhesion is required for effect. In addition, IDO activity has been shown to induce monocytes’ differentiation towards IL-10, producing immunosuppressive CD14 + CD206+ macrophages (alternatively activated M2 cells), and thus limiting T cell activation and proliferation. Moreover, IDO dependent pathways play a role in CD5+ regulatory B cell (Breg) induction [128]. Similarly to IDO, NO has been proposed as an essential factor in regulating T cell responses that may require direct interaction of MSC and T cells [129]. Furthermore, MSCs involve oxidative stress pathways through inducible cytoprotective enzyme heme oxygenase 1 (HO-1) that catalyzes the heme to biliverdin, which in turn suppresses T cell proliferation [130].

Similarly to small molecules, MSC-derived anti-inflammatory cytokines, namely IL-10, IL-35, and TGFβ, play a central role in indirect immune regulatory mechanisms. The anti-inflammatory properties of IL-10 were recognized as a central mechanism of MSC-mediated regulation of innate and adaptive immune compartments. Its function is associated with: downregulation of Th1 and Th17 derived cytokines [94], regulation of HLA-DR, CD80, and CD86 expression on APC and thus induction of their tolerogenic phenotype [131, 132], blocking of NF-κB signaling [133], regulation of IL-1α, IL-1β [134], IL-12p40 [135], IFNγ [136], and TNF-α production [134], among others. Notably, by induction of regulatory T cells and alternatively activated macrophages, MSCs indirectly enhance IL-10 release [93, 137, 138]. In some contrast to IL-10, IL-35 represents a relatively new described cytokine belonging to the IL-12 family [139]; thus, its function remains not fully elucidated. Immune regulatory properties of IL-35 are associated with the selective expansion of Treg and a decrease of Th17 immune response [140]. Moreover, MSC-derived IL-35 promotes the conversion of B cells to IL-10 producing Bregs [141]. However, further studies are needed to better understand the importance of MSC-derived IL-35 in immune regulation, healing, and regeneration. On the other hand, TGFβ may act as both a potent regulator or an activator of innate and adaptive immune responses [142]. Its function depends on the composition of local activating factors (recently reviewed elsewhere [143]). However, it seems that MSC-derived TGFβ contributes to the polarization of activated T cells towards Tregs and promotes their expansion. In addition, recently, TGFβ induced plasticity of Th17 cells towards regulatory phenotype was reported [144]; however, the mechanism remains elusive. In macrophages, TGFβ was shown to regulate NF-κB signaling and thus control their inflammatory response. Moreover, it polarizes monocytes towards M2 alternatively activated cells [145]. On the other hand, however, TGFβ signaling has been found to play a role in lung fibrosis and promotion of Th17 cells in which other MSC-derived factors may further induce functional plasticity [142, 146]. However, complex interplay between different protein and non-protein components of MSC-derived secretome and their effects on immune function needs more attention in the future.

In the past two decades, an additional cellular communication mechanism that involves the transfer of extracellular vesicles (EVs) has been proposed as a soluble factor-dependent mechanism [3, 147, 148]. EVs are classified according to their cellular origins into exosomes (endocytic bodies in the size of 30–150 nm), microvesicles (vehicles derived from the budding of the cell membrane in the size of 100–1000 nm), and apoptotic bodies (500–5000 nm) [148–150]. Recently, it became clear that EVs represent an important component of MSC-derived secretome. Both exosomes and microvesicles show overlapping characteristics and may shuttle functional proteins, lipids, and nucleic acids (including mRNAs, miRNAs, and lncRNAs) with immune-modulatory properties. In fact, over 900 different proteins have been currently recognized in MSC-derived EVs according to the exosome database, available at: http://www.exocarta.org. EVs are characterized by the presence of surface CD9, CD29, CD44, CD63, CD73, CD81,CD105, and CD107. Immune regulatory properties are linked especially to growth factors and cytokine (TGFβ, IL-6, IL-10, and HGF) [96, 132, 151–154], enzymes (IDO) [155], lipid mediators (PGE-2) [156, 157], and miRNAs (miR-155, miR-146, and miR-594) [158]. It seems, however, that the content depends on the activation and the source of MSCs. MSC-derived EVs were shown to i) decrease IL-1β and TNF-α expression in glial cells [159], ii) regulate T cell responses, and increase Treg proliferation [160], iii) regulate DC maturation [161, 162], and iv) suppress mast cell activation [163]. Interestingly, it appeared that the beneficial effects of MSC-derived EVs are comparable to entire MSCs. Therefore, they are increasingly recognized as a potential therapeutic factor for inflammatory and degenerative diseases. However, to date, the effects of MSC-derived EVs in the regulation of asthmatic inflammation remain elusive.

## Mechanisms of MSC Mediated Regulation of Asthmatic Lung Inflammation

**Fig. 2:**
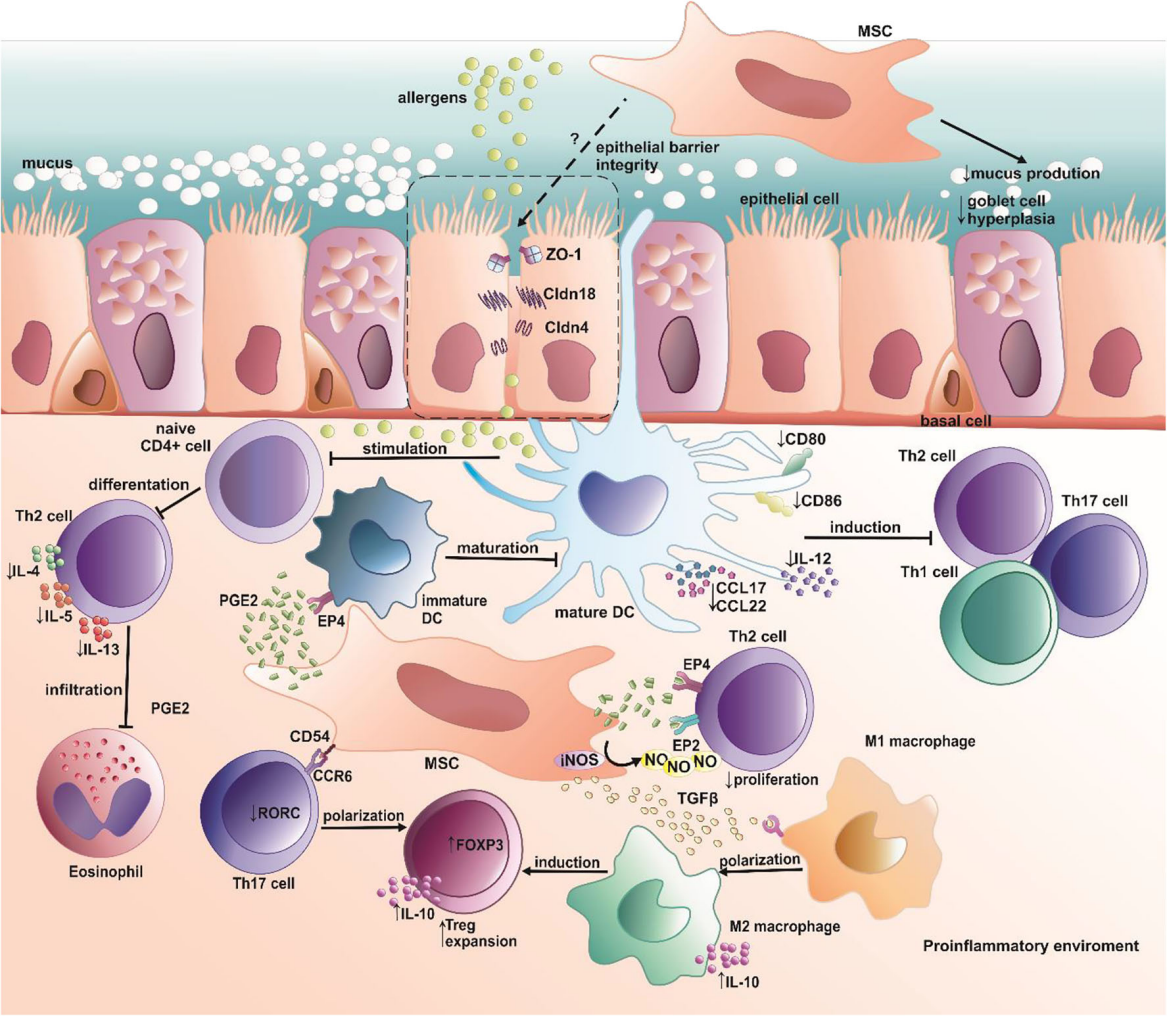
Mesenchymal stem cells-mediated immunomodulatory effects in the airways. Summary of immunomodulatory effects of mesenchymal stem cells in asthmatic lung inflammation. The set of proinflammatory cytokines secreted in the course of asthma causes the priming of mesenchymal stem cells and induces their immunosuppressive activities. Importantly, induction of regulatory T cells, alternatively activated macrophages (M2), and tolerogenic dendritic cells (DCs) represent to date best-described mechanisms regulating Th2-driven and non-Th2-driven immune responses. CCL(17/22)- chemokine C-C motif ligand 17/22; CCR6- C-C chemokine receptor 6; Cldn(4/18)- claudin 4/18; MSC- mesenchymal stem cell; ZO-1- zonula occludens 1; DC- dendritic cells; EP(2/4)- prostaglandin E2 receptor 2/4; FOXP3- forkhead box p3; IL-(4, 5, 10, 12, 13, 35)- interleukin 4, 5, 10, 12, 13, 35; iNOS- inducible nitric oxide synthase; NO- nitric oxide, PGE2- prostaglandin E2; RORC- RAR-related orphan receptor gamma; TGFβ- transforming growth factor β, ←activation/ induction; ├ inhibition; ← - unknown effect; ↑increase; ↓decrease

Asthmatic airway inflammation should be considered a complex network of interactions between different lung resident cells, immune cells, growth factors, enzymes, cytokines, chemokines, metabolites, and miRNAs [164]. Unfortunately, our understanding of the effects of MSC on this network is significantly restricted due to limitations of used models, namely xenotransplantation models of human MSC into mice or usage of mice cells only **(**Table 1**).** However, in this section, we summarize the current understanding of MSC effects on each of the lung’s crucial inflammatory cascade components (Fig. 2).

**Table 1:** Mesenchymal stem cell-mediated effects in different experimental asthma models

Source and number of MSC	Route of MSC administration	Type of experimental asthma model	Mice strain	Histological outcome and respiratory mechanics	Immunomodulatory outcome or proposed mechanism	Reference
Mice ADMSCs (0,3 × 10^6^ cells)	Intravenously	Intranasal challenge with HDM extract (100 μg/nare)	BALB/c	Ø AHR ↓ airway responsiveness ↓ immune cell infiltration ↓total leukocytes number in BALF ↓ goblet cells hyperplasia ↓airway contractile tissue remodeling ↓extracellular matrix mass ↓eosinophilia in BALF, whereas greater after BMMSCs administration	↓ IgE in BALF ↑ IFNγ, IL-12, FGFb in BALF	[35]
MCA-MSCs from a clinical-grade iPSC line (1 × 10^6^ cells)	Intravenously (IN), intranasally (IV)	Intraperitoneal injection with 10 μg OVA and 400 μg of potassium alum adjuvant; nebulization with aerosolized OVA (2,5% in NaCl)	Balb/c	↓ peribronchial inflammatory cell infiltration (IN, IV) ↓goblet cells (IN, IV) ↓collagen deposition and concentration (IN, IV); whereas IN to a greater extent ↓subepithelial myofibroblast density (IN, IV); whereas IN to a greater extent ↓airway epithelial thickness (only IN)	↓ TGFβ (IN, IV) ↑MMP-9 (IN, IV), whereas IN to a greater extent ↓AHR (IV partially, IN completely)	[88]
Mice BMMSCs (0,5 × 10^6^ cells)	Intravenously	Intraperitoneal injection of 100 μg/ml OVA in aluminum hydroxide; intranasally challenge with 50 μg/ml of OVA in PBS	BALB/cOlaHsd (H-2d)	↓peribronchial inflammation ↓ bronchial hyperreactivity compared ↓airway mucus secretion ↓airway eosinophilia ↓macrophages count in BALF	↓allergen-specific IgE immune response ↓IL4-, IL-13 in BALF and restimulated spleen cells ↑ IL-10 in BALF in BALF and restimulated spleen cells ↑ CD4 + CD25 + Foxp3+ cells in lungs and spleens; Tregs dependent mechanism	[165]
Mice ADMSCs (1 × 10^6^ cells)	Intravenously	Intraperitoneal injection of 75 μg of OVA in 2 mg of aluminum hydroxide in 200 μL PBS; an intranasal challenge with 1 μg/ μL of OVA (in PBS)	C57BL/6	↓the occurrence of nasal symptoms ↓inflammatory cells and eosinophils count in BALF ↓goblet cell hyperplasia	↓OVA-specific IgE, IgG1, IgG1/IgG2 ratio ↑OVA-specific IgG2 ↑CD4 + CD25 and IFNγ+CD4+ cells in mLNs ↓IL-4 + CD4+ cells in mLN ↑IDO and TGFβ gene in lung tissue ↑PGE2 gene in serum	[175]
Mice BMMSCs (0,5 × 10^6^ cells)	Intravenously	Percutaneous injection with 25 μg/μl of Der f extract in DMSO; an intranasal challenge with 6,25 μg/μl of Der f extract in PBS	Balb/c	↓AHR ↓bronchoconstriction ↓airway inflammation ↓neutrophils, eosinophils, lymphocytes count in BAL	↑M2 muscarinic receptor expression Ø M3 muscarinic receptor expression ↓IL-4, IL-5, IL-13, IL-17 by lung CD4+ cells ØIFNγ and IL-10 by lung CD4+ cells ↑M2 alveolar macrophage phenotype through COX2/ PGE2 dependent signaling	[177]
Human ADMSCs (0,1 × 10^6^ cells) and their EVs (37 μg)	Intravenously	Intraperitoneal injection with 1 μg/μl of OVA (adjuvant-free, in saline); intratracheal administration with 1 μg/μl of OVA (in saline)	C57BL/6	ADMSC- derived EVs mediated effect and ADMSC: ↓collagen deposition in the lung parenchyma and airway ↓total leukocyte number in BALF ↓eosinophils count in BALF ADMSC- derived EVs mediated effect: ↑respiratory mechanics ↑eosinophils in lung tissue ADMSCs mediated effect: Ø respiratory mechanics Ø eosinophils number in lung tissue	ADMSC- derived EVs mediated effect and ADMSC: ↓TGFβ in lung tissue Ø IFNγ and IL-10 in BALF ↓CD3 + CD4+ T cells in the thymus Ø CD3 + CD4+ in mLNs ADMSC- derived EVs mediated effect: ↓CD3 + CD4+ T cells in BALF ↓IL-5, IL-13 in BALF ADMSCs mediated effect: ↓CD4 + CD25 + Foxp3+ in BALF ↓IL-5, IL-13, eotaxin in BALF	[178]
Mice BMMSCs (1 × 10^6^ cells)	Intravenously	Intransally induction with 0,5 μg/μl and 0,1 μg/μl of HDM extract	BALB/c (H2d)	In the acute model: ↓airway lymphocytes, neutrophils, eosinophils, monocytes count ↓mucus secretion ↓peribronchial eosinophilia, the mast cells count ↓AHR In the chronic model: ↓airway lymphocytes, neutrophils, eosinophils count ↓mucus secretion	In the acute model: ↓total IgE in serum ↓IL-5, IL-13 in BALF Ø IFNγ in BALF Ø ILCs recruitment In the chronic model: ↓total IgE in serum ↓ IL-13 in BALF ↓ CD11b + DCs in lungs ↓ MHCII CD86+ DCs in lungs Ø DCs in mLNs ↓IL-25, IL-1α in the lung	[179]
Mice ADMSCs (1 × 10^6^ cells)	IntravenousIy	Intraperitoneal injection with 0,1% OVA (in PBS); challenge with aerosolized 2.5% OVA (in PBS)	BALB/c	↓ AHR ↓inflammatory cells number in the lungs ↓mucus-producing goblet cells, Muc5ac secretion ↓total cells, eosinophils, and lymphocytes count in BALF	↓total IgE in serum ↓IL-4, IL-17F in BALF ↑IL-10, IFNγ in BALF ↑CD4 + CD25 + Foxp3 cells in spleen	[180]
Human UCMSCs (0,3 × 10^6^, 0,6 × 10^6^ cells)	Intravenously	Intranasal sensitization with 75 μg OVA and 10 μg Poly(I:C); Intranasal challenge with 50 μg OVA and 10 μg Poly(I:C)	BALB/c	↓airway inflammation ↓total number of cells and neutrophils in BALF	↓IL-5 and CXCL15 in BALF Ø IL-10, IFNγ in BALF ↓IL-5, IL-17, IFNγ in mLNs Ø IL-10 in mLNs	[181]
BMMSCs, ADMSCs, LMSCs (0,1 × 10^6^ cells)	Intratracheally	Intraperitoneal injection with OVA 0,1 μg/μl (in saline); intratracheal administration with 1 μg/μl of OVA (in saline)	C57BL/6	↑lung mechanics, whereas greater extent to BMMSCs ↓inflammatory cell infiltration in lung tissue ↓ the alveolar collapse in the lung parenchyma (only after BMMSCs administration) ↓collagen fiber content (only after BMMSCs)	↓ IL-4 and IL-13 in lung tissue homogenates, whereas greater after BMMSC and ADMSC compared to LMSCs ↑IL-10 in lung tissue homogenates and whereas greater after BMMSC and ADMSC compared to LMSCs ↓TGFβ in lung tissue homogenates, comparable for all MSC types ↓VEGF in lung tissue homogenates, whereas greater after BMMSC	[182]
Human BMMSCs (1 × 10^6^ cells)	Intravenously	Intraperitoneal injection with 10 μg of OVA in 1.5 mg of Al(OH)3 (100 μg total volume); an intranasal challenge with 1% weight/volume OVA in PBS	BALB/c	↓ total number of cells in BALF ↓ epithelial cell thickening ↓ mucus production, goblet cells hyperplasia ↓ collagen cell deposition	↓systemic IgE ↓IFNγ, IL-5, IL-13 in BALF ØTNF-α, IL-6 in BALF ↑ MIP-1α and KC in BALF ↓iNOS in the lungs	[183]
Human ADMSCs, UCMSCs BMMSCs (1 × 10^6^ cells)	Intravenously	Intraperitoneal injection with 50 μg OVA with 2 mg aluminum hydroxide gel; intranasal administration with 50 μg OVA	Balb/c	ADMSC-, UCMSC- and BMMSC-mediated effect: ↓AHR ↓mucus-producing goblet cells number ↓inflammatory cells in airway tissue ↓eosinophils count in BALF	ADMSC-, UCMSC- and BMMSC-mediated effect: ↓IL-5 and IL-13 by mLNs T cells ØIFNγ by mLNs T cells ↑proportion and absolute number of alveolar macrophages ADMSC mediated effect: ↓IL-4 by bronchial and mLNs T cells ↓IL-5 in BALF and lungs Ø M1/M2 switching in lungs Ø TGFβ in lungs macrophages ØIL-10 in lung macrophages	[187]
Mice ADMSCs (0,2 × 10^6^ cells)	Intravenously	Intraperitoneal injection with 20 μg OVA with 1 mg of alum as an adjuvant; nebulization OVA (1% in saline)	C57BL/6 (WT; IFN-γ−/−; IFN-γR−/−; CCL2−/−)	↓AHR ↓airway eosinophilia ↓eosinophils count in BALF ↓goblet cell metaplasia	↓IL-4, IL-5, IL-13 in BALF ↑IL-10 in BALF ↑CCR2+ monocytes recruitment to the lung Ø CD4+ and CD8+ T cells in the lung tissue Ø CD11b + Ly6G+ neutrophils in the lung tissue Ø B cells in lung tissue Ø NK cells in lung tissue ↑ IL-10 producing monocytes/macrophages depend on IFNγ primed CCL2/CCR2 pathway in lung tissue	[188]
Mice BMMSCs (2 × 10^6^ cells)	Intravenously	Intraperitoneal injection with 100 mg of OVA in 9% aluminum hydroxide hydrate; Intratracheal challenged with 100 mg OVA (in PBS)	C57BL/6	↓immune cells infiltration, eosinophilia ↓peribronchial inflammation and eosinophilia ↓AHR	↑ CXCR4/SDF-1 axis dependent migration of MSC to lungs ↓ mast cell mediator (β-hexosaminidase) and mast cell degranulation ↓IL-4, IL-5, IL-9 in BALF ↑IFNγ, and IFNγ/ IL-4 (mRNA) ratio; shift from Th2 to Th1 response	[189]
Mice BMMSCs and ADMSCs (0,1 × 10^6^ cells)	Intratracheally	Intranasal induction with 1 μg/μl of HDM extract in PBS	C57BL/6	BMMSC-mediated effect: ↓lung elastance (only in combination with methacholine) ↓ eosinophils, macrophages, and neutrophils count in BALF ADMSC-mediated effect: Ø lung elastance and airway resistance ↓ macrophages and neutrophils count in BALF ADMSC- and BMMSC-mediated effect: Ø presence of lung inflammatory pockets and mucus-filled cells	BMMSC-mediated effect: ↑IL-10 in lung homogenate Ø CD4+ IL-10 producing cells ↓IL-1β, IL-6 in alveolar macrophages ↓ B cells in mLNs ↓ CD4+ cells in mLNs ADMSC- and BMMSC-mediated effect: Ø CCL11, CCL24, IL-4, IL-5, TGFβ in lung homogenate Ø CD4+ cells in BALF ↓number of CD4 + CD25 + Foxp3+ cells in BALF Ø CD4 + CD25 + Foxp3+ cells in mLNs	[191]
Human BMMSCs (1 × 10^6^ cells)	Intravenously	Intraperitoneal injection with 50 μg OVA with 2 mg aluminum hydroxide gel; an intranasal challenge with 50 μg OVA	NOD/SCID	↓AHR ↓eosinophilia in BALF	Ø macrophage recruitment ↑polarization of CD163- M1 macrophages toward CD163+ M2 macrophages through TGFβ dependent signaling	[204]
Mice BMMSCs (0,25 × 10^6^ cells)	Intravenously	Intraperitoneal sensitization with 10 μg/0.1 ml OVA in alum adjuvant; a challenge with 1% aerosolized OVA (in PBS) via airways	BALB/c	↓immune cells infiltration in the lungs ↓ goblet cells number ↓ epithelium the smooth muscle layer, basement membrane thickness	↑ CD4 + CD25 + Foxp3 cells frequency in T lymphocytes in lungs	[212]
Mice BMMSC (0,5 × 10^6^ cells)	Intratracheally	Intraperitoneal injection with 100 μg OVA in 1.3 mg aluminum hydroxide (in 200 μl); nebulization with 2.5% OVA (in PBS)	Balb/c	↓neutrophils, eosinophils, monocytes count in BALF ↓airway inflammation, goblet cells hyperplasia, subepithelial fibrosis	↑IL-12 in serum and BALF ↓IL-4 in serum and BALF Ø IL-10, IFNγ, IL-13 ↑ratio CD4 + CD25+ cells/ lymphocytes in pulmonary lymph nodes	[211]

ADMSCs- adipose tissue-derived mesenchymal stem cells; AHR-airway hyperresponsiveness; BALF- bronchoalveolar lavage fluid; BMMSCs- bone marrow-derived mesenchymal stem cells; CD(4,8, 11b, 25, 163)- custer of differentation 4, 8, 11b, 25, 163; CCL(2, 11, 24)- chemokine (CC motif) ligand 2, 11, 24; CCR2- CC chemokine receptor type 2; COX2- cyclooyxgenase 2; CXCL15- chemokine (CXC motif) ligand 15; CXCR4- CXC chemokine receptor type 4; DCs- dendritic cells; EVs- extracellular vesicles; FGFb- basic fibroblast growth factor; Foxp3- forkhead box p3; HDM- house dust mite; IDO- indoleamine 2,3-dioxygenase; IFNγ- interferon gamma; Ig(E, G1, G2)- immunoglobulin E, G1, G2; IL-(1a, 4, 5, 6, 9, 10, 12, 13, 17, 25)-interleukin 1a, 4, 5, 6, 9, 10, 13, 17, 25; ILCs- innate lymphoid cells; iNOS- inducible nitric oxide synthase; Ly6G- lymphocyte antigen 6 complex locus G6D; KC- keratinocyte derived chemokine; LMSCs- lung derived mesenchymal stem cells; MCA-MSCs- mesenchymoangioblast-derived mesenchymal stem cells; MHCII- major histocompabillity complex class II; PBS- phosphate buffered saline; MIP-1α- macrophage infammatory protein 1α; mLNs- mediastinal lymph nodes; MMP9- matrix metalloproteinase 9; OVA- Ovalbumin; SDF-1- stromal-derived factor 1; TGFβ- transforming growth factor-beta; TNF-α- tumor necrosis; factor-alpha; UCMSCs- umbilical cord blood-derived mesenchymal stem cells; VEGF- vascular endothelial growth factor; WT- wild type, Ø no effect observed; ↑an increase / improvement; ↓a decrease/ limitation

To date, MSCs-mediated effects on T cell function are the best characterized and are associated with the regulation of their proliferation and functional plasticity [165, 166]. T cell proliferation leads to the formation of high numbers of effector cells [167]. Activated MSCs regulate this process by the production of nitric oxide (NO) and PGE2 [168, 169]. NO production in MSC is controlled by inducible nitric oxide synthase (iNOS) [170]. This pathway increases NO levels, which induces S-nitrosylation of transcription factors, metabolic enzymes, and cytoskeletal proteins [171]. In some contrast, PGE2 promotes T cell anergy by the regulation of IL-2 production and IL-2R (CD25) expression on activated cells [117, 172]. In addition, PGE2 has been demonstrated to suppress Th1 differentiation and enhance the induction and differentiation of adaptive regulatory T cells in the lungs [94].

Although T cells acquire their effector function towards antigen presentation by antigen-presenting cells (APC) upon TCR and costimulatory molecule engagement, they may retain functional plasticity and acquire additional cytokine-producing capacities upon re-stimulation [173]. Interestingly, MSC may directly induce functional plasticity by the epigenetic reprogramming of Th17 cells. In fact, the interaction between IFNγ and TNF-α activated MSCs and Th17 cells via CD56 – CCR6 (CD196) receptors induces IL-10 production and histone H3K4me3 trimethylation in the FOXP3 locus promoter with subsequent suppression of RAR-related orphan receptor C (RORC) [174]. Consequently, Th17 cells lose their immune-activatory properties and acquire suppressive (regulatory) functions. Furthermore, MSCs have been shown to regulate the expression of Th2 cytokines, namely IL-4, IL-5, and IL-13 [36, 175–183]. However, it needs to be addressed whether observed regulation may represent a consequence of T cell plasticity or is a consequence of Th2 cell anergy and in consequence apoptosis. Notably, a manifestation of disease symptoms considered extensively as hallmarks of asthma is directly or indirectly related to the overproduction of IL-4, IL-5, and IL-13 [184, 185]. More specifically, IL-4 synergistically with IL-13 induces the antibody class switching towards immunoglobulin E (IgE), produced by B cells [184], whereas IL-5 plays a key role in the survival, differentiation, degranulation, and recruitment of eosinophils to the site of inflammation [184, 186]. In mice that lack IL-4, IL-5, IL-13 reduction of asthma symptoms was observed in the Ova-Alum experimental model. Thus, administration of MSC may block the initial steps of allergic sensitization cascade through regulation of Th2-related cytokines [165, 177, 178, 187–189]. Nevertheless, it became clear that activated MSC may enhance regulatory T cell activity and induce the production of anti-inflammatory cytokines, namely IL-10 and TGFβ, in both direct and indirect mechanisms. It seems that soluble factor-mediated mechanisms are sufficient to induce regulatory effector functions of Treg, while direct cell-to-cell interaction is needed for their expansion [36, 165, 178, 190, 191]. Nonetheless, the mechanistic of MSC-Treg interactions remain poorly understood and are likely to be complex and dependent on the local lower airway microenvironment.

Dendritic cells (DCs) are referred to as a professional antigen-presenting cell (APC) linking innate and adaptive immune responses. Therefore, they are recognized as central players in the inflammatory cascade [192, 193]. It seems that MSC may directly regulate DC maturation and differentiation from monocytes and CD34+ progenitors through PGE2 dependent mechanism [194]. Notably, immature or semi-mature DCs possess tolerogenic properties and may regulate T cells’ proinflammatory responses and induce Treg maturation. In contrast to immature DCs, mature cells from asthmatic patients present high expression of costimulatory molecules, namely CD80 and CD86, and possess high T cell stimulatory properties [132, 195]. In fact, they are involved in the polarization of T cell responses towards Th1/Th17 or Th2 cells and thus may support both eosinophilic and non-eosinophilic (neutrophilic) lung inflammation [164, 180, 181, 193]. Importantly, however, MSCs were shown to decrease the above-described properties by regulation of DC expression of costimulatory molecules and proinflammatory cytokine secretion. In addition, MSC decreases the release of CC chemokine ligand (CCL)17 and CCL22 chemokines by dendritic cells and, thus, regulate T cell responses within the lung [196].

Similarly to DCs, macrophages possess antigen-presenting capacities, and due to their pleiotropic biological activities, they may orchestrate both adaptive and innate immune responses. Macrophages can be polarized towards two distinct phenotypes, namely M1 (classically activated) and M2 (alternatively activated) cells [197–200]. M1 macrophages are recognized as immune stimulatory cells producing high amounts of proinflammatory cytokines (including TNF-α, IL-1, IL-6, IL-12, and IL-23), chemokines (such as CCL5, CCL8, CXCL2, and CXCL4), polarizing T cell responses towards Th1 and Th17, and possess high antigen presentation capacity. In contrast, M2 macrophages possess immune regulatory/reparatory properties with high secretion of anti-inflammatory IL-10, IL-35, TGFβ, IL-1 receptor antagonist (IL-1RA), CCL16, CCL18, and CCL22, the ability for induction of regulatory T cells, and lack of cytotoxic activity [187, 201–203]. However, due to their capability to support Th2 response, their role in allergic lung inflammation remains not fully elucidated. Interestingly, MSCs were shown to support macrophages’ alternative activation in the IL-10 and/or TGFβ related mechanism [98, 204]. In fact, an accumulating body of evidence indicates the central role of alveolar macrophages in the MSCs-mediated immunosuppression in the asthmatic lung. It seems that the MSC-induced M2-derived immunosuppression supports induction of regulatory T cells within the lungs [204]. However, the mechanisms of MSC-macrophage mutual interactions need to be elucidated in the future.

Airway epithelial cells play a central role in innate immune function as the first line of defense against biological, physical, and chemical stressors. Moreover, activated epithelial cells play a central role in inflammatory cascade by releasing inflammatory mediators, namely cytokines and chemokines [193, 205, 206]. The function of epithelial barrier depends directly on the expression of proteins building tight connections between epithelial cells referred to as tight junction (TJ) proteins [186]. Disruption of their function is currently recognized as a hallmark of asthma [184, 185, 207]. Moreover, differentially regulated expression of tight junction related genes may be observed in distinct asthma phenotypes [186]. More specifically, reduced zonula occludens-1 (Zo-1) and Claudin 18 expression are typical for all asthma phenotypes, while the upregulation of Claudin 4 and Claudin 7 seems to be specific only to neutrophilic airway inflammation [186, 208]. By some contrast, Claudin 1, Claudin 5, and Claudin 7 expression were downregulated only in the eosinophilic phenotype [186]. Thus, the personalized targeting of particular tight junction proteins may be a useful therapeutic option for individual asthma phenotypes. In fact, impairment of epithelial integrity, observed in asthma, results in barrier leaking, leading to the intensified inflammatory response [205, 206]. Unfortunately, to date, the influence of the MSCs on the epithelial barrier function remains elusive. Notably, disrupted epithelial barrier integrity and uncontrolled lung inflammation may partially contribute to the development of the combination of irreversible structural changes within the lung tissue referred to as airway remodeling [209]. Mucus overproduction, smooth muscle hyperplasia, and increased collagen deposition, resulting in airflow obstruction contribute to the clinical manifestation of a disease. Although some of the concepts explain airway remodeling development to some degree, to date, available therapies do not allow to alleviate its progress [210]. However, several studies reported that MSC administration attenuates airway remodeling by limiting goblet cell hyperplasia, epithelial thickness, subepithelial smooth muscle hyperplasia, and inhibit collagen deposition [35, 211–214]. Notably, the mechanism underlying the mentioned beneficial effects of MSCs remains elusive. Increased deposition of collagen fibers (collagen I, III, V, XI) to extracellular matrix seems to be strongly related to the secretion of profibrotic factor from eosinophils, such as TGFβ [215, 216]. In addition, TGFβ promotes the release of matrix metalloproteinase 9 (MMP-9), which subsequently contributes to extend collagen deposition [217]. Nevertheless, the loss or augmentation of MMPs activity, also through inadequate control by their tissue inhibitors (TIMPs) results in fibrosis development. Thus, the maintaining or restoring of a balance between MMPs and TIMPs activity seems to prevent remodeling development [218]. Notably, both intranasal and intravenous administration of MSCs causes the increased activity of MMP-9 suggesting activation of compensatory processes [88]. Interestingly, TGFβ may act as a regulator of MSCs mediated immunosuppression; however, MSCs decrease the level of TGFβ within the lung tissue and thus may limit lung remodeling [204]. Therefore, implementation of mesenchymal stem cells may hold great promise for preventing pathological tissue reconstructions observed in inflammed lung remodeling. Unfortunately, the mechanism underlying the mentioned beneficial effects of MSCs remains elusive and need further research.

## Conclusions

The accumulating body of evidence confirms the beneficial effects of MSCs in different preclinical asthma models. The vast majority of published studies utilized the xenotransplantation of human MSC into mice. Notably, the model possesses critical weaknesses resulting from some of the MSC-derived mediators’ restricted species function. On the other hand, however, mice MSCs may not fully cover the human MSC function. Indeed, these issues represent a significant obstacle to understanding the mechanisms by which MSCs regulate asthmatic lung inflammation and postpone their use in clinical practice. However, according to the clinical trials database (http://clinicaltrials.gov) first clinical trial was performed to assess the safety, tolerability, and efficacy of allogeneic human mesenchymal stem cells infusion in a total of 6 patients with mild asthma [219]. The study was terminated as the first cohort was completed. Unfortunately, to date, the results are not yet available.

In summary, we do not claim that stem cell-based therapies should or will replace currently used effective first-line treatment in asthma. However, stem cells can become an attractive and relatively safe option for helping those patients who failed to satisfactorily respond to conventional treatment.

## References

[1] Trounson A, McDonald C (2015). Stem cell therapies in clinical trials: Progress and challenges. Cell Stem Cell.

[2] Rezania A, Bruin JE, Arora P, Rubin A, Batushansky I, Asadi A, O'Dwyer S, Quiskamp N, Mojibian M, Albrecht T, Yang YHC, Johnson JD, Kieffer TJ (2014). Reversal of diabetes with insulin-producing cells derived in vitro from human pluripotent stem cells. Nature Biotechnology.

[3] Ratajczak MZ, Kucia M, Jadczyk T, Greco NJ, Wojakowski W, Tendera M, Ratajczak J (2012). Pivotal role of paracrine effects in stem cell therapies in regenerative medicine: Can we translate stem cell-secreted paracrine factors and microvesicles into better therapeutic strategies?. Leukemia.

[4] Parmar M, Grealish S, Henchcliffe C (2020). The future of stem cell therapies for Parkinson disease. Nature Reviews. Neuroscience.

[5] Madl CM, Heilshorn SC, Blau HM (2018). Bioengineering strategies to accelerate stem cell therapeutics. Nature.

[6] Guha P, Morgan JW, Mostoslavsky G, Rodrigues NP, Boyd AS (2013). Lack of immune response to differentiated cells derived from syngeneic induced pluripotent stem cells. Cell Stem Cell.

[7] Itakura G, Kawabata S, Ando M, Nishiyama Y, Sugai K, Ozaki M, Iida T, Ookubo T, Kojima K, Kashiwagi R, Yasutake K, Nakauchi H, Miyoshi H, Nagoshi N, Kohyama J, Iwanami A, Matsumoto M, Nakamura M, Okano H (2017). Fail-safe system against potential Tumorigenicity after transplantation of iPSC derivatives. Stem Cell Reports.

[8] Zhao T, Zhang ZN, Rong Z, Xu Y (2011). Immunogenicity of induced pluripotent stem cells. Nature.

[9] Araki R, Uda M, Hoki Y, Sunayama M, Nakamura M, Ando S, Sugiura M, Ideno H, Shimada A, Nifuji A, Abe M (2013). Negligible immunogenicity of terminally differentiated cells derived from induced pluripotent or embryonic stem cells. Nature.

[10] Huang XP, Sun Z, Miyagi Y, McDonald Kinkaid H, Zhang L, Weisel RD, Li RK (2010). Differentiation of allogeneic mesenchymal stem cells induces immunogenicity and limits their long-term benefits for myocardial repair. Circulation.

[11] Rama P, Matuska S, Paganoni G, Spinelli A, De Luca M, Pellegrini G (2010). Limbal stem-cell therapy and long-term corneal regeneration. The New England Journal of Medicine.

[12] Lee JS, Hong JM, Moon GJ, Lee PH, Ahn YH, Bang OY, STARTING collaborators (2010). A long-term follow-up study of intravenous autologous mesenchymal stem cell transplantation in patients with ischemic stroke. Stem Cells.

[13] Martin RM, Fowler JL, Cromer MK, Lesch BJ, Ponce E, Uchida N, Nishimura T, Porteus MH, Loh KM (2020). Improving the safety of human pluripotent stem cell therapies using genome-edited orthogonal safeguards. Nature Communications.

[14] Approved Cellular and Gene Therapy Products (2020). https://www.fda.gov/vaccines-blood-biologics/cellular-gene-therapy-products/approved-cellular-and-gene-therapy-products. Accessed December 9, 2020.

[15] First stem-cell therapy recommended for approval in EU (2014). https://www.ema.europa.eu/en/news/first-stem-cell-therapy-recommended-approval-eu. Accessed December 9, 2020.

[16] Martin I, Galipeau J, Kessler C, Le Blanc K, Dazzi F (2019). Challenges for mesenchymal stromal cell therapies. Science Translational Medicine.

[17] De Luca M, Aiuti A, Cossu G, Parmar M, Pellegrini G, Robey PG (2019). Advances in stem cell research and therapeutic development. Nature Cell Biology.

[18] Kay AG, Long G, Tyler G, Stefan A, Broadfoot SJ, Piccinini AM, Middleton J, Kehoe O (2017). Mesenchymal stem cell-conditioned medium reduces disease severity and immune responses in inflammatory arthritis. Scientific Reports.

[19] Murphy KC, Whitehead J, Falahee PC, Zhou D, Simon SI, Leach JK (2017). Multifactorial experimental design to optimize the anti-inflammatory and Proangiogenic potential of Mesenchymal stem cell spheroids. Stem Cells.

[20] Redondo-Castro E, Cunningham C, Miller J, Martuscelli L, Aoulad-Ali S, Rothwell NJ, Kielty CM, Allan SM, Pinteaux E (2017). Interleukin-1 primes human mesenchymal stem cells towards an anti-inflammatory and pro-trophic phenotype in vitro. Stem Cell Research & Therapy.

[21] Luger D, Lipinski MJ, Westman PC, Glover DK, Dimastromatteo J, Frias JC, Albelda MT, Sikora S, Kharazi A, Vertelov G, Waksman R, Epstein SE (2017). Intravenously delivered Mesenchymal stem cells: Systemic anti-inflammatory effects improve left ventricular dysfunction in acute myocardial infarction and ischemic cardiomyopathy. Circulation Research.

[22] Park SR, Kim JW, Jun HS, Roh JY, Lee HY, Hong IS (2018). Stem cell Secretome and its effect on Cellular mechanisms relevant to wound healing. Molecular Therapy.

[23] Tachibana A, Santoso MR, Mahmoudi M, Shukla P, Wang L, Bennett M, Goldstone AB, Wang M, Fukushi M, Ebert AD, Woo YJ, Rulifson E, Yang PC (2017). Paracrine effects of the pluripotent stem cell-derived cardiac Myocytes salvage the injured myocardium. Circulation Research.

[24] Li X, Xie X, Lian W, Shi R, Han S, Zhang H, Lu L, Li M (2018). Exosomes from adipose-derived stem cells overexpressing Nrf2 accelerate cutaneous wound healing by promoting vascularization in a diabetic foot ulcer rat model. Experimental & Molecular Medicine.

[25] de Mendonça L, Felix NS, Blanco NG, Da Silva JS, Ferreira TP, Abreu SC (2017). Mesenchymal stromal cell therapy reduces lung inflammation and vascular remodeling and improves hemodynamics in experimental pulmonary arterial hypertension. Stem Cell Research & Therapy.

[26] Uemura R, Xu M, Ahmad N, Ashraf M (2006). Bone marrow stem cells prevent left ventricular remodeling of ischemic heart through paracrine signaling. Circulation Research.

[27] Lan YW, Choo KB, Chen CM, Hung TH, Chen YB, Hsieh CH, Kuo HP, Chong KY (2015). Hypoxia-preconditioned mesenchymal stem cells attenuate bleomycin-induced pulmonary fibrosis. Stem Cell Research & Therapy.

[28] Horton JA, Hudak KE, Chung EJ, White AO, Scroggins BT, Burkeen JF, Citrin DE (2013). Mesenchymal stem cells inhibit cutaneous radiation-induced fibrosis by suppressing chronic inflammation. Stem Cells.

[29] Wilson JG, Liu KD, Zhuo H, Caballero L, McMillan M, Fang X, Cosgrove K, Vojnik R, Calfee CS, Lee JW, Rogers AJ, Levitt J, Wiener-Kronish J, Bajwa EK, Leavitt A, McKenna D, Thompson BT, Matthay MA (2015). Mesenchymal stem (stromal) cells for treatment of ARDS: A phase 1 clinical trial. The Lancet Respiratory Medicine.

[30] Glassberg MK, Minkiewicz J, Toonkel RL, Simonet ES, Rubio GA, DiFede D, Shafazand S, Khan A, Pujol MV, LaRussa VF, Lancaster LH, Rosen GD, Fishman J, Mageto YN, Mendizabal A, Hare JM (2017). Allogeneic human Mesenchymal stem cells in patients with idiopathic pulmonary fibrosis via intravenous delivery (AETHER): A phase I safety clinical trial. Chest.

[31] Gu W, Song L, Li XM, Wang D, Guo XJ, Xu WG (2015). Mesenchymal stem cells alleviate airway inflammation and emphysema in COPD through down-regulation of cyclooxygenase-2 via p38 and ERK MAPK pathways. Scientific Reports.

[32] Li X, Michaeloudes C, Zhang Y, Wiegman CH, Adcock IM, Lian Q (2018). Mesenchymal stem cells alleviate oxidative stress-induced mitochondrial dysfunction in the airways. The Journal of Allergy and Clinical Immunology.

[33] Hao Q, Gudapati V, Monsel A, Park JH, Hu S, Kato H, Lee JH, Zhou L, He H, Lee JW (2019). Mesenchymal stem cell-derived extracellular vesicles decrease lung injury in mice. Journal of Immunology.

[34] Ahn SY, Park WS, Kim YE, Sung DK, Sung SI, Ahn JY, Chang YS (2018). Vascular endothelial growth factor mediates the therapeutic efficacy of mesenchymal stem cell-derived extracellular vesicles against neonatal hyperoxic lung injury. Experimental & Molecular Medicine.

[35] Mariñas-Pardo L, Mirones I, Amor-Carro O, Fraga-Iriso R, Lema-Costa B, Cubillo I, Rodríguez Milla MÁ, García-Castro J, Ramos-Barbón D (2014). Mesenchymal stem cells regulate airway contractile tissue remodeling in murine experimental asthma. Allergy.

[36] Kapoor S, Patel SA, Kartan S, Axelrod D, Capitle E, Rameshwar P (2012). Tolerance-like mediated suppression by mesenchymal stem cells in patients with dust mite allergy-induced asthma. The Journal of Allergy and Clinical Immunology.

[37] Akdis CA (2012). Therapies for allergic inflammation: Refining strategies to induce tolerance. Nature Medicine.

[38] Barnes PJ (2012). Severe asthma: Advances in current management and future therapy. The Journal of Allergy and Clinical Immunology.

[39] Corren J (2019). New targeted therapies for uncontrolled asthma. The Journal of Allergy and Clinical Immunology. In Practice.

[40] Agache I, Rocha C, Beltran J, Song Y, Posso M, Solà I, Alonso-Coello P, Akdis C, Akdis M, Canonica GW, Casale T, Chivato T, Corren J, del Giacco S, Eiwegger T, Firinu D, Gern JE, Hamelmann E, Hanania N, Mäkelä M, Martín IH, Nair P, O'Mahony L, Papadopoulos NG, Papi A, Park HS, Pérez de Llano L, Quirce S, Sastre J, Shamji M, Schwarze J, Canelo-Aybar C, Palomares O, Jutel M (2020). Efficacy and safety of treatment with biologicals (benralizumab, dupilumab and omalizumab) for severe allergic asthma: A systematic review for the EAACI guidelines - recommendations on the use of biologicals in severe asthma. Allergy.

[41] Agache I, Lau S, Akdis CA, Smolinska S, Bonini M, Cavkaytar O, Flood B, Gajdanowicz P, Izuhara K, Kalayci O, Mosges R, Palomares O, Papadopoulos NG, Sokolowska M, Angier E, Fernandez-Rivas M, Pajno G, Pfaar O, Roberts GC, Ryan D, Sturm GJ, Ree R, Varga EM, Wijk RG, Yepes-Nuñez JJ, Jutel M (2019). EAACI guidelines on allergen immunotherapy: House dust mite-driven allergic asthma. Allergy.

[42] Corren J, Castro M, O'Riordan T, Hanania NA, Pavord ID, Quirce S, Chipps BE, Wenzel SE, Thangavelu K, Rice MS, Harel S, Jagerschmidt A, Khan AH, Kamat S, Maroni J, Rowe P, Lu Y, Amin N, Pirozzi G, Ruddy M, Graham NMH, Teper A (2020). Dupilumab efficacy in patients with uncontrolled, moderate-to-severe allergic asthma. The Journal of Allergy and Clinical Immunology. In Practice.

[43] Caplan AI (1991). Mesenchymal stem cells. Journal of Orthopaedic Research.

[44] Pittenger MF, Mackay AM, Beck SC, Jaiswal RK, Douglas R, Mosca JD, Moorman MA, Simonetti DW, Craig S, Marshak DR (1999). Multilineage potential of adult human mesenchymal stem cells. Science.

[45] Caplan AI (2017). Mesenchymal stem cells: Time to change the name!. Stem Cells Translational Medicine.

[46] Han SM, Han SH, Coh YR, Jang G, Chan Ra J, Kang SK, Lee HW, Youn HY (2014). Enhanced proliferation and differentiation of Oct4- and Sox2-overexpressing human adipose tissue mesenchymal stem cells. Experimental & Molecular Medicine.

[47] Krampera M, Glennie S, Dyson J, Scott D, Laylor R, Simpson E, Dazzi F (2003). Bone marrow mesenchymal stem cells inhibit the response of naive and memory antigen-specific T cells to their cognate peptide. Blood.

[48] Glennie S, Soeiro I, Dyson PJ, Lam EW, Dazzi F (2005). Bone marrow mesenchymal stem cells induce division arrest anergy of activated T cells. Blood.

[49] Wang HS, Hung SC, Peng ST, Huang CC, Wei HM, Guo YJ, Fu YS, Lai MC, Chen CC (2004). Mesenchymal stem cells in the Wharton's jelly of the human umbilical cord. Stem Cells.

[50] Lee OK, Kuo TK, Chen WM, Lee KD, Hsieh SL, Chen TH (2004). Isolation of multipotent mesenchymal stem cells from umbilical cord blood. Blood.

[51] Bieback K, Kern S, Klüter H, Eichler H (2004). Critical parameters for the isolation of mesenchymal stem cells from umbilical cord blood. Stem Cells.

[52] Roubelakis MG, Pappa KI, Bitsika V, Zagoura D, Vlahou A, Papadaki HA, Antsaklis A, Anagnou NP (2007). Molecular and proteomic characterization of human mesenchymal stem cells derived from amniotic fluid: Comparison to bone marrow mesenchymal stem cells. Stem Cells and Development.

[53] Savickiene J, Treigyte G, Baronaite S, Valiuliene G, Kaupinis A, Valius M, Arlauskiene A, Navakauskiene R (2015). Human amniotic fluid Mesenchymal stem cells from second- and third-trimester amniocentesis: Differentiation potential, molecular signature, and proteome analysis. Stem Cells International.

[54] Tsai MS, Hwang SM, Chen KD, Lee YS, Hsu LW, Chang YJ, Wang CN, Peng HH, Chang YL, Chao AS, Chang SD, Lee KD, Wang TH, Wang HS, Soong YK (2007). Functional network analysis of the transcriptomes of mesenchymal stem cells derived from amniotic fluid, amniotic membrane, cord blood, and bone marrow. Stem Cells.

[55] Shi S, Gronthos S (2003). Perivascular niche of postnatal mesenchymal stem cells in human bone marrow and dental pulp. Journal of Bone and Mineral Research.

[56] Schüring AN, Schulte N, Kelsch R, Röpke A, Kiesel L, Götte M (2011). Characterization of endometrial mesenchymal stem-like cells obtained by endometrial biopsy during routine diagnostics. Fertility and Sterility.

[57] Meng X, Ichim TE, Zhong J, Rogers A, Yin Z, Jackson J, Wang H, Ge W, Bogin V, Chan KW, Thébaud B, Riordan NH (2007). Endometrial regenerative cells: a novel stem cell population. Journal of Translational Medicine.

[58] Tondreau T, Meuleman N, Delforge A, Dejeneffe M, Leroy R, Massy M, Mortier C, Bron D, Lagneaux L (2005). Mesenchymal stem cells derived from CD133-positive cells in mobilized peripheral blood and cord blood: Proliferation, Oct4 expression, and plasticity. Stem Cells.

[59] Wang SJ, Jiang D, Zhang ZZ, Huang AB, Qi YS, Wang HJ, Zhang JY, Yu JK (2016). Chondrogenic potential of peripheral blood derived Mesenchymal stem cells seeded on demineralized Cancellous bone scaffolds. Scientific Reports.

[60] Xu J, Su Y, Hu L, Cain A, Gu Y, Liu B, Wu R, Wang S, Wang H (2018). Effect of bone morphogenetic protein 6 on Immunomodulatory functions of salivary gland-derived Mesenchymal stem cells in Sjögren's syndrome. Stem Cells and Development.

[61] de Sousa EB, Casado PL, Moura Neto V, Duarte ME, Aguiar DP (2014). Synovial fluid and synovial membrane mesenchymal stem cells: Latest discoveries and therapeutic perspectives. Stem Cell Research & Therapy.

[62] Liu TM, Martina M, Hutmacher DW, Hui JH, Lee EH, Lim B (2007). Identification of common pathways mediating differentiation of bone marrow- and adipose tissue-derived human mesenchymal stem cells into three mesenchymal lineages. Stem Cells.

[63] Viswanathan S, Shi Y, Galipeau J, Krampera M, Leblanc K, Martin I, Nolta J, Phinney DG, Sensebe L (2019). Mesenchymal stem versus stromal cells: International Society for Cell & gene therapy (ISCT®) Mesenchymal stromal cell committee position statement on nomenclature. Cytotherapy.

[64] Mattar P, Bieback K (2015). Comparing the Immunomodulatory properties of bone marrow, adipose tissue, and birth-associated tissue Mesenchymal stromal cells. Frontiers in Immunology.

[65] Ribeiro A, Laranjeira P, Mendes S, Velada I, Leite C, Andrade P, Santos F, Henriques A, Grãos M, Cardoso CMP, Martinho A, Pais ML, da Silva C, Cabral J, Trindade H, Paiva A (2013). Mesenchymal stem cells from umbilical cord matrix, adipose tissue and bone marrow exhibit different capability to suppress peripheral blood B, natural killer and T cells. Stem Cell Research & Therapy.

[66] Najar M, Raicevic G, Boufker HI, Fayyad Kazan H, De Bruyn C, Meuleman N (2010). Mesenchymal stromal cells use PGE2 to modulate activation and proliferation of lymphocyte subsets: Combined comparison of adipose tissue, Wharton's jelly and bone marrow sources. Cellular Immunology.

[67] Ivanova-Todorova E, Bochev I, Mourdjeva M, Dimitrov R, Bukarev D, Kyurkchiev S, Tivchev P, Altunkova I, Kyurkchiev DS (2009). Adipose tissue-derived mesenchymal stem cells are more potent suppressors of dendritic cells differentiation compared to bone marrow-derived mesenchymal stem cells. Immunology Letters.

[68] Jin HJ, Bae YK, Kim M, Kwon SJ, Jeon HB, Choi SJ (2013). Comparative analysis of human mesenchymal stem cells from bone marrow, adipose tissue, and umbilical cord blood as sources of cell therapy. International Journal of Molecular Sciences.

[69] Li X, Bai J, Ji X, Li R, Xuan Y, Wang Y (2014). Comprehensive characterization of four different populations of human mesenchymal stem cells as regards their immune properties, proliferation and differentiation. International Journal of Molecular Medicine.

[70] Adams TS, Schupp JC, Poli S, Ayaub EA, Neumark N, Ahangari F (2020). Single-cell RNA-seq reveals ectopic and aberrant lung-resident cell populations in idiopathic pulmonary fibrosis. Science Advances.

[71] Habermann AC, Gutierrez AJ, Bui LT, Yahn SL, Winters NI, Calvi CL (2020). Single-cell RNA sequencing reveals profibrotic roles of distinct epithelial and mesenchymal lineages in pulmonary fibrosis. Science Advances.

[72] Wang L, Shi M, Tong L, Wang J, Ji S, Bi J, Chen C, Jiang J, Bai C, Zhou J, Song Y (2019). Lung-resident Mesenchymal stem cells promote repair of LPS-induced acute lung injury via regulating the balance of regulatory T cells and Th17 cells. Inflammation.

[73] Tong L, Zhou J, Rong L, Seeley EJ, Pan J, Zhu X, Liu J, Wang Q, Tang X, Qu J, Bai C, Song Y (2016). Fibroblast growth Factor-10 (FGF-10) mobilizes lung-resident Mesenchymal stem cells and protects against acute lung injury. Scientific Reports.

[74] Lu Z, Chang W, Meng S, Xu X, Xie J, Guo F, Yang Y, Qiu H, Liu L (2019). Mesenchymal stem cells induce dendritic cell immune tolerance via paracrine hepatocyte growth factor to alleviate acute lung injury. Stem Cell Research & Therapy.

[75] Rolandsson Enes S, Andersson Sjöland A, Skog I, Hansson L, Larsson H, Le Blanc K (2016). MSC from fetal and adult lungs possess lung-specific properties compared to bone marrow-derived MSC. Scientific Reports.

[76] Cao H, Wang C, Chen X, Hou J, Xiang Z, Shen Y, Han X (2018). Inhibition of Wnt/β-catenin signaling suppresses myofibroblast differentiation of lung resident mesenchymal stem cells and pulmonary fibrosis. Scientific Reports.

[77] Cao H, Chen X, Hou J, Wang C, Xiang Z, Shen Y, Han X (2020). The Shh/Gli signaling cascade regulates myofibroblastic activation of lung-resident mesenchymal stem cells via the modulation of Wnt10a expression during pulmonary fibrogenesis. Laboratory Investigation.

[78] Shi C, Cao X, Chen X, Sun Z, Xiang Z, Zhao H, Qian W, Han X (2015). Intracellular surface-enhanced Raman scattering probes based on TAT peptide-conjugated au nanostars for distinguishing the differentiation of lung resident mesenchymal stem cells. Biomaterials.

[79] Hynes K, Menicanin D, Mrozik K, Gronthos S, Bartold PM (2014). Generation of functional mesenchymal stem cells from different induced pluripotent stem cell lines. Stem Cells and Development.

[80] Tang M, Chen W, Liu J, Weir MD, Cheng L, Xu HH (2014). Human induced pluripotent stem cell-derived mesenchymal stem cell seeding on calcium phosphate scaffold for bone regeneration. Tissue Engineering. Part A.

[81] Pinto DS, Ahsan T, Serra J, Fernandes-Platzgummer A, Cabral JMS, da Silva CL (2020). Modulation of the in vitro angiogenic potential of human mesenchymal stromal cells from different tissue sources. Journal of Cellular Physiology.

[82] Soontararak S, Chow L, Johnson V, Coy J, Wheat W, Regan D, Dow S (2018). Mesenchymal stem cells (MSC) derived from induced pluripotent stem cells (iPSC) equivalent to adipose-derived MSC in promoting intestinal healing and microbiome normalization in mouse inflammatory bowel disease model. Stem Cells Translational Medicine.

[83] Yoshihara M, Hayashizaki Y, Murakawa Y (2017). Genomic instability of iPSCs: Challenges towards their clinical applications. Stem Cell Reviews and Reports.

[84] A Study of CYP-001 for the Treatment of Steroid-Resistant Acute Graft Versus Host Disease (2016). https://clinicaltrials.gov/ct2/show/NCT02923375?term=cyp-001&draw=2&rank=1. Accessed December 9, 2020.

[85] The MEseNchymal coviD-19 Trial: a Pilot Study to Investigate Early Efficacy of MSCs in Adults With COVID-19 (MEND) (2020). https://clinicaltrials.gov/ct2/show/NCT04537351?term=cyp-001&draw=2&rank=2. Accessed December 9, 2020.

[86] Yao Y, Fan XL, Jiang D, Zhang Y, Li X, Xu ZB, Fang SB, Chiu S, Tse HF, Lian Q, Fu QL (2018). Connexin 43-mediated mitochondrial transfer of iPSC-MSCs alleviates asthma inflammation. Stem Cell Reports.

[87] Royce SG, Mao W, Lim R, Kelly K, Samuel CS (2019). iPSC- and mesenchymoangioblast-derived mesenchymal stem cells provide greater protection against experimental chronic allergic airways disease compared with a clinically used corticosteroid. The FASEB Journal.

[88] Royce SG, Rele S, Broughton BRS, Kelly K, Samuel CS (2017). Intranasal administration of mesenchymoangioblast-derived mesenchymal stem cells abrogates airway fibrosis and airway hyperresponsiveness associated with chronic allergic airways disease. The FASEB Journal.

[89] Ren G, Zhang L, Zhao X, Xu G, Zhang Y, Roberts AI, Zhao RC, Shi Y (2008). Mesenchymal stem cell-mediated immunosuppression occurs via concerted action of chemokines and nitric oxide. Cell Stem Cell.

[90] Sudres M, Norol F, Trenado A, Grégoire S, Charlotte F, Levacher B, Lataillade JJ, Bourin P, Holy X, Vernant JP, Klatzmann D, Cohen JL (2006). Bone marrow mesenchymal stem cells suppress lymphocyte proliferation in vitro but fail to prevent graft-versus-host disease in mice. Journal of Immunology.

[91] Zappia E, Casazza S, Pedemonte E, Benvenuto F, Bonanni I, Gerdoni E, Giunti D, Ceravolo A, Cazzanti F, Frassoni F, Mancardi G, Uccelli A (2005). Mesenchymal stem cells ameliorate experimental autoimmune encephalomyelitis inducing T-cell anergy. Blood.

[92] Zhou Y, Day A, Haykal S, Keating A, Waddell TK (2013). Mesenchymal stromal cells augment CD4+ and CD8+ T-cell proliferation through a CCL2 pathway. Cytotherapy.

[93] Deng Y, Zhang Y, Ye L, Zhang T, Cheng J, Chen G, Zhang Q, Yang Y (2016). Umbilical cord-derived Mesenchymal stem cells instruct monocytes towards an IL10-producing phenotype by secreting IL6 and HGF. Scientific Reports.

[94] Luz-Crawford P, Kurte M, Bravo-Alegría J, Contreras R, Nova-Lamperti E, Tejedor G, Noël D, Jorgensen C, Figueroa F, Djouad F, Carrión F (2013). Mesenchymal stem cells generate a CD4+CD25+Foxp3+ regulatory T cell population during the differentiation process of Th1 and Th17 cells. Stem Cell Research & Therapy.

[95] Sheng H, Wang Y, Jin Y, Zhang Q, Zhang Y, Wang L, Shen B, Yin S, Liu W, Cui L, Li N (2008). A critical role of IFNgamma in priming MSC-mediated suppression of T cell proliferation through up-regulation of B7-H1. Cell Research.

[96] Spaggiari GM, Capobianco A, Becchetti S, Mingari MC, Moretta L (2006). Mesenchymal stem cell-natural killer cell interactions: Evidence that activated NK cells are capable of killing MSCs, whereas MSCs can inhibit IL-2-induced NK-cell proliferation. Blood.

[97] Spaggiari GM, Capobianco A, Abdelrazik H, Becchetti F, Mingari MC, Moretta L (2008). Mesenchymal stem cells inhibit natural killer-cell proliferation, cytotoxicity, and cytokine production: Role of indoleamine 2,3-dioxygenase and prostaglandin E2. Blood.

[98] Luz-Crawford P, Djouad F, Toupet K, Bony C, Franquesa M, Hoogduijn MJ, Jorgensen C, Noël D (2016). Mesenchymal stem cell-derived interleukin 1 receptor antagonist promotes macrophage polarization and inhibits B cell differentiation. Stem Cells.

[99] Rosado MM, Bernardo ME, Scarsella M, Conforti A, Giorda E, Biagini S, Cascioli S, Rossi F, Guzzo I, Vivarelli M, dello Strologo L, Emma F, Locatelli F, Carsetti R (2015). Inhibition of B-cell proliferation and antibody production by mesenchymal stromal cells is mediated by T cells. Stem Cells and Development.

[100] Zhou H, Guo M, Bian C, Sun Z, Yang Z, Zeng Y, Ai HS, Zhao RC (2010). Efficacy of bone marrow-derived mesenchymal stem cells in the treatment of sclerodermatous chronic graft-versus-host disease: Clinical report. Biology of Blood and Marrow Transplantation.

[101] Duijvestein M, Vos AC, Roelofs H, Wildenberg ME, Wendrich BB, Verspaget HW (2010). Autologous bone marrow-derived mesenchymal stromal cell treatment for refractory luminal Crohn’s disease: Results of a phase I study. Gut.

[102] Wang D, Li J, Zhang Y, Zhang M, Chen J, Li X, Hu X, Jiang S, Shi S, Sun L (2014). Umbilical cord mesenchymal stem cell transplantation in active and refractory systemic lupus erythematosus: A multicenter clinical study. Arthritis Research & Therapy.

[103] Kim Y, Jin HJ, Heo J, Ju H, Lee HY, Kim S, Lee S, Lim J, Jeong SY, Kwon JH, Kim M, Choi SJ, Oh W, Yang YS, Hwang HH, Yu HY, Ryu CM, Jeon HB, Shin DM (2018). Small hypoxia-primed mesenchymal stem cells attenuate graft-versus-host disease. Leukemia.

[104] Waterman RS, Tomchuck SL, Henkle SL, Betancourt AM (2010). A new mesenchymal stem cell (MSC) paradigm: Polarization into a pro-inflammatory MSC1 or an immunosuppressive MSC2 phenotype. PLoS One.

[105] Lee SC, Jeong HJ, Lee SK, Kim SJ (2015). Lipopolysaccharide preconditioning of adipose-derived stem cells improves liver-regenerating activity of the secretome. Stem Cell Research & Therapy.

[106] Noone C, Kihm A, English K, O'Dea S, Mahon BP (2013). IFN-gamma stimulated human umbilical-tissue-derived cells potently suppress NK activation and resist NK-mediated cytotoxicity in vitro. Stem Cells and Development.

[107] Francois M, Romieu-Mourez R, Li M, Galipeau J (2012). Human MSC suppression correlates with cytokine induction of indoleamine 2,3-dioxygenase and bystander M2 macrophage differentiation. Molecular Therapy.

[108] Sivanathan KN, Rojas-Canales DM, Hope CM, Krishnan R, Carroll RP, Gronthos S, Grey ST, Coates PT (2015). Interleukin-17A-induced human Mesenchymal stem cells are superior modulators of immunological function. Stem Cells.

[109] Benvenuto F, Ferrari S, Gerdoni E, Gualandi F, Frassoni F, Pistoia V, Mancardi G, Uccelli A (2007). Human mesenchymal stem cells promote survival of T cells in a quiescent state. Stem Cells.

[110] Pevsner-Fischer M, Morad V, Cohen-Sfady M, Rousso-Noori L, Zanin-Zhorov A, Cohen S, Cohen IR, Zipori D (2007). Toll-like receptors and their ligands control mesenchymal stem cell functions. Blood.

[111] Gao F, Chiu SM, Motan DA, Zhang Z, Chen L, Ji HL (2016). Mesenchymal stem cells and immunomodulation: Current status and future prospects. Cell Death & Disease.

[112] Fang SB, Zhang HY, Wang C, He BX, Liu XQ, Meng XC, Peng YQ, Xu ZB, Fan XL, Wu ZJ, Chen D, Zheng L, Zheng SG, Fu QL (2020). Small extracellular vesicles derived from human mesenchymal stromal cells prevent group 2 innate lymphoid cell-dominant allergic airway inflammation through delivery of miR-146a-5p. J Extracell Vesicles.

[113] Li Y, Zhang D, Xu L, Dong L, Zheng J, Lin Y, Huang J, Zhang Y, Tao Y, Zang X, Li D, du M (2019). Cell-cell contact with proinflammatory macrophages enhances the immunotherapeutic effect of mesenchymal stem cells in two abortion models. Cellular & Molecular Immunology.

[114] Ni K, Liu M, Zheng J, Wen L, Chen Q, Xiang Z, Lam KT, Liu Y, Chan GCF, Lau YL, Tu W (2018). PD-1/PD-L1 pathway mediates the alleviation of pulmonary fibrosis by human Mesenchymal stem cells in humanized mice. American Journal of Respiratory Cell and Molecular Biology.

[115] Li H, Wang W, Wang G, Hou Y, Xu F, Liu R, Wang F, Xue J, Hu T, Luan X (2015). Interferon-γ and tumor necrosis factor-α promote the ability of human placenta-derived mesenchymal stromal cells to express programmed death ligand-2 and induce the differentiation of CD4(+)interleukin-10(+) and CD8(+)interleukin-10(+)Treg subsets. Cytotherapy.

[116] Singh AK, Stock P, Akbari O (2011). Role of PD-L1 and PD-L2 in allergic diseases and asthma. Allergy.

[117] Wang WB, Yen ML, Liu KJ, Hsu PJ, Lin MH, Chen PM, Sudhir PR, Chen CH, Chen CH, Sytwu HK, Yen BL (2015). Interleukin-25 mediates transcriptional control of PD-L1 via STAT3 in multipotent human Mesenchymal stromal cells (hMSCs) to suppress Th17 responses. Stem Cell Reports.

[118] Azevedo RI, Minskaia E, Fernandes-Platzgummer A, Vieira AIS, da Silva CL, Cabral JMS, Lacerda JF (2020). Mesenchymal stromal cells induce regulatory T cells via epigenetic conversion of human conventional CD4 T cells in vitro. Stem Cells.

[119] Lee HJ, Kim SN, Jeon MS, Yi T, Song SU (2017). ICOSL expression in human bone marrow-derived mesenchymal stem cells promotes induction of regulatory T cells. Scientific Reports.

[120] Krampera M, Cosmi L, Angeli R, Pasini A, Liotta F, Andreini A, Santarlasci V, Mazzinghi B, Pizzolo G, Vinante F, Romagnani P, Maggi E, Romagnani S, Annunziato F (2006). Role for interferon-gamma in the immunomodulatory activity of human bone marrow mesenchymal stem cells. Stem Cells.

[121] Gieseke F, Böhringer J, Bussolari R, Dominici M, Handgretinger R, Müller I (2010). Human multipotent mesenchymal stromal cells use galectin-1 to inhibit immune effector cells. Blood.

[122] Chinnadurai R, Copland IB, Patel SR, Galipeau J (2014). IDO-independent suppression of T cell effector function by IFN-γ-licensed human mesenchymal stromal cells. Journal of Immunology.

[123] Ren G, Zhao X, Zhang L, Zhang J, L'Huillier A, Ling W, Roberts AI, le AD, Shi S, Shao C, Shi Y (2010). Inflammatory cytokine-induced intercellular adhesion molecule-1 and vascular cell adhesion molecule-1 in mesenchymal stem cells are critical for immunosuppression. Journal of Immunology.

[124] Schena F, Gambini C, Gregorio A, Mosconi M, Reverberi D, Gattorno M, Casazza S, Uccelli A, Moretta L, Martini A, Traggiai E (2010). Interferon-γ-dependent inhibition of B cell activation by bone marrow-derived mesenchymal stem cells in a murine model of systemic lupus erythematosus. Arthritis and Rheumatism.

[125] Luk F, Carreras-Planella L, Korevaar SS, de Witte SFH, Borràs FE, Betjes MGH, Baan CC, Hoogduijn MJ, Franquesa M (2017). Inflammatory conditions dictate the effect of Mesenchymal stem or stromal cells on B cell function. Frontiers in Immunology.

[126] Luk F, de Witte SF, Korevaar SS, Roemeling-van Rhijn M, Franquesa M, Strini T (2016). Inactivated Mesenchymal stem cells maintain Immunomodulatory capacity. Stem Cells and Development.

[127] Li W, Ren G, Huang Y, Su J, Han Y, Li J, Chen X, Cao K, Chen Q, Shou P, Zhang L, Yuan ZR, Roberts AI, Shi S, le AD, Shi Y (2012). Mesenchymal stem cells: A double-edged sword in regulating immune responses. Cell Death and Differentiation.

[128] Peng Y, Chen X, Liu Q, Zhang X, Huang K, Liu L, Li H, Zhou M, Huang F, Fan Z, Sun J, Liu Q, Ke M, Li X, Zhang Q, Xiang AP (2015). Mesenchymal stromal cells infusions improve refractory chronic graft versus host disease through an increase of CD5+ regulatory B cells producing interleukin 10. Leukemia.

[129] Kim J, Hematti P (2009). Mesenchymal stem cell-educated macrophages: A novel type of alternatively activated macrophages. Experimental Hematology.

[130] Mougiakakos D, Jitschin R, Johansson CC, Okita R, Kiessling R, Le Blanc K (2011). The impact of inflammatory licensing on heme oxygenase-1-mediated induction of regulatory T cells by human mesenchymal stem cells. Blood.

[131] Li YP, Paczesny S, Lauret E, Poirault S, Bordigoni P, Mekhloufi F, Hequet O, Bertrand Y, Ou-Yang JP, Stoltz JF, Miossec P, Eljaafari A (2008). Human mesenchymal stem cells license adult CD34+ hemopoietic progenitor cells to differentiate into regulatory dendritic cells through activation of the notch pathway. Journal of Immunology.

[132] Djouad F, Charbonnier LM, Bouffi C, Louis-Plence P, Bony C, Apparailly F, Cantos C, Jorgensen C, Noël D (2007). Mesenchymal stem cells inhibit the differentiation of dendritic cells through an interleukin-6-dependent mechanism. Stem Cells.

[133] Zhong Z, Chen A, Fa Z, Ding Z, Xiao L, Wu G, Wang Q, Zhang R (2020). Bone marrow mesenchymal stem cells upregulate PI3K/AKT pathway and down-regulate NF-κB pathway by secreting glial cell-derived neurotrophic factors to regulate microglial polarization and alleviate deafferentation pain in rats. Neurobiology of Disease.

[134] Sgrignoli MR, Silva DA, Nascimento FF, Sgrignoli DAM, Nai GA, da Silva MG, de Barros MA, Bittencourt MKW, de Morais BP, Dinallo HR, Foglia BTD, Cabrera WB, Fares EC, Andrade SF (2019). Reduction in the inflammatory markers CD4, IL-1, IL-6 and TNFα in dogs with keratoconjunctivitis sicca treated topically with mesenchymal stem cells. Stem Cell Research.

[135] Shi B, Qi J, Yao G, Feng R, Zhang Z, Wang D, Chen C, Tang X, Lu L, Chen W, Sun L (2018). Mesenchymal stem cell transplantation ameliorates Sjögren's syndrome via suppressing IL-12 production by dendritic cells. Stem Cell Research & Therapy.

[136] Chatterjee D, Marquardt N, Tufa DM, Hatlapatka T, Hass R, Kasper C, von Kaisenberg C, Schmidt RE, Jacobs R (2014). Human umbilical cord-derived Mesenchymal stem cells utilize Activin-a to suppress interferon-γ production by natural killer cells. Frontiers in Immunology.

[137] Abumaree MH, Al Jumah MA, Kalionis B, Jawdat D, Al Khaldi A, Abomaray FM (2013). Human placental mesenchymal stem cells (pMSCs) play a role as immune suppressive cells by shifting macrophage differentiation from inflammatory M1 to anti-inflammatory M2 macrophages. Stem Cell Reviews and Reports.

[138] Lim JY, Im KI, Lee ES, Kim N, Nam YS, Jeon YW, Cho SG (2016). Enhanced immunoregulation of mesenchymal stem cells by IL-10-producing type 1 regulatory T cells in collagen-induced arthritis. Scientific Reports.

[139] Dong J, Wong CK, Cai Z, Jiao D, Chu M, Lam CW (2015). Amelioration of allergic airway inflammation in mice by regulatory IL-35 through dampening inflammatory dendritic cells. Allergy.

[140] Whitehead GS, Wilson RH, Nakano K, Burch LH, Nakano H, Cook DN (2012). IL-35 production by inducible costimulator (ICOS)-positive regulatory T cells reverses established IL-17-dependent allergic airways disease. The Journal of Allergy and Clinical Immunology.

[141] Cho KA, Lee JK, Kim YH, Park M, Woo SY, Ryu KH (2017). Mesenchymal stem cells ameliorate B-cell-mediated immune responses and increase IL-10-expressing regulatory B cells in an EBI3-dependent manner. Cellular & Molecular Immunology.

[142] O'Garra A, Stockinger B, Veldhoen M (2008). Differentiation of human T(H)-17 cells does require TGF-beta!. Nature Immunology.

[143] Batlle E, Massagué J (2019). Transforming growth factor-β signaling in immunity and cancer. Immunity.

[144] Zhou L, Lopes JE, Chong MM, Ivanov II, Min R, Victora GD (2008). TGF-beta-induced Foxp3 inhibits T(H)17 cell differentiation by antagonizing RORgammat function. Nature.

[145] Gong D, Shi W, Yi SJ, Chen H, Groffen J, Heisterkamp N (2012). TGFβ signaling plays a critical role in promoting alternative macrophage activation. BMC Immunology.

[146] Paun A, Bergeron ME, Haston CK (2017). The Th1/Th17 balance dictates the fibrosis response in murine radiation-induced lung disease. Scientific Reports.

[147] Ratajczak J, Miekus K, Kucia M, Zhang J, Reca R, Dvorak P, Ratajczak MZ (2006). Embryonic stem cell-derived microvesicles reprogram hematopoietic progenitors: Evidence for horizontal transfer of mRNA and protein delivery. Leukemia.

[148] Ratajczak J, Wysoczynski M, Hayek F, Janowska-Wieczorek A, Ratajczak MZ (2006). Membrane-derived microvesicles: Important and underappreciated mediators of cell-to-cell communication. Leukemia.

[149] Ratajczak MZ, Ratajczak J (2020). Extracellular microvesicles/exosomes: Discovery, disbelief, acceptance, and the future?. Leukemia.

[150] Phinney DG, Di Giuseppe M, Njah J, Sala E, Shiva S, St Croix CM (2015). Mesenchymal stem cells use extracellular vesicles to outsource mitophagy and shuttle microRNAs. Nature Communications.

[151] Kim HS, Choi DY, Yun SJ, Choi SM, Kang JW, Jung JW, Hwang D, Kim KP, Kim DW (2012). Proteomic analysis of microvesicles derived from human mesenchymal stem cells. Journal of Proteome Research.

[152] Du T, Zou X, Cheng J, Wu S, Zhong L, Ju G (2013). Human Wharton's jelly-derived mesenchymal stromal cells reduce renal fibrosis through induction of native and foreign hepatocyte growth factor synthesis in injured tubular epithelial cells. Stem Cell Research & Therapy.

[153] Aggarwal S, Pittenger MF (2005). Human mesenchymal stem cells modulate allogeneic immune cell responses. Blood.

[154] Di Nicola M, Carlo-Stella C, Magni M, Milanesi M, Longoni PD, Matteucci P (2002). Human bone marrow stromal cells suppress T-lymphocyte proliferation induced by cellular or nonspecific mitogenic stimuli. Blood.

[155] Hwu P, Du MX, Lapointe R, Do M, Taylor MW, Young HA (2000). Indoleamine 2,3-dioxygenase production by human dendritic cells results in the inhibition of T cell proliferation. Journal of Immunology.

[156] Harting MT, Srivastava AK, Zhaorigetu S, Bair H, Prabhakara KS, Toledano Furman NE, Vykoukal JV, Ruppert KA, Cox CS, Olson SD (2018). Inflammation-stimulated Mesenchymal stromal cell-derived extracellular vesicles attenuate inflammation. Stem Cells.

[157] Wu S, Ju GQ, Du T, Zhu YJ, Liu GH (2013). Microvesicles derived from human umbilical cord Wharton's jelly mesenchymal stem cells attenuate bladder tumor cell growth in vitro and in vivo. PLoS One.

[158] Di Trapani M, Bassi G, Midolo M, Gatti A, Kamga PT, Cassaro A (2016). Differential and transferable modulatory effects of mesenchymal stromal cell-derived extracellular vesicles on T, B and NK cell functions. Scientific Reports.

[159] Thomi G, Surbek D, Haesler V, Joerger-Messerli M, Schoeberlein A (2019). Exosomes derived from umbilical cord mesenchymal stem cells reduce microglia-mediated neuroinflammation in perinatal brain injury. Stem Cell Research & Therapy.

[160] Zhu H, Lan L, Zhang Y, Chen Q, Zeng Y, Luo X, Ren J, Chen S, Xiao M, Lin K, Chen M, Li Q, Chen Y, Xu J, Zheng Z, Chen Z, Xie Y, Hu J, Yang T (2020). Epidermal growth factor stimulates exosomal microRNA-21 derived from mesenchymal stem cells to ameliorate aGVHD by modulating regulatory T cells. The FASEB Journal.

[161] Reis M, Mavin E, Nicholson L, Green K, Dickinson AM, Wang XN (2018). Mesenchymal stromal cell-derived extracellular vesicles attenuate dendritic cell maturation and function. Frontiers in Immunology.

[162] Shahir M, Mahmoud Hashemi S, Asadirad A, Varahram M, Kazempour-Dizaji M, Folkerts G, Garssen J, Adcock I, Mortaz E (2020). Effect of mesenchymal stem cell-derived exosomes on the induction of mouse tolerogenic dendritic cells. Journal of Cellular Physiology.

[163] Cho BS, Kim JO, Ha DH, Yi YW (2018). Exosomes derived from human adipose tissue-derived mesenchymal stem cells alleviate atopic dermatitis. Stem Cell Research & Therapy.

[164] Lambrecht BN, Hammad H (2015). The immunology of asthma. Nature Immunology.

[165] Kavanagh H, Mahon BP (2011). Allogeneic mesenchymal stem cells prevent allergic airway inflammation by inducing murine regulatory T cells. Allergy.

[166] Chen QH, Wu F, Liu L, Chen HB, Zheng RQ, Wang HL, Yu LN (2020). Mesenchymal stem cells regulate the Th17/Treg cell balance partly through hepatocyte growth factor in vitro. Stem Cell Research & Therapy.

[167] Boonpiyathad T, Sokolowska M, Morita H, Rückert B, Kast JI, Wawrzyniak M, Sangasapaviliya A, Pradubpongsa P, Fuengthong R, Thantiworasit P, Sirivichayakul S, Kwok WW, Ruxrungtham K, Akdis M, Akdis CA (2019). Der p 1-specific regulatory T-cell response during house dust mite allergen immunotherapy. Allergy.

[168] Rasmusson I, Ringdén O, Sundberg B, Le Blanc K (2005). Mesenchymal stem cells inhibit lymphocyte proliferation by mitogens and alloantigens by different mechanisms. Experimental Cell Research.

[169] Sato K, Ozaki K, Oh I, Meguro A, Hatanaka K, Nagai T, Muroi K, Ozawa K (2007). Nitric oxide plays a critical role in suppression of T-cell proliferation by mesenchymal stem cells. Blood.

[170] Su J, Chen X, Huang Y, Li W, Li J, Cao K, Cao G, Zhang L, Li F, Roberts AI, Kang H, Yu P, Ren G, Ji W, Wang Y, Shi Y (2014). Phylogenetic distinction of iNOS and IDO function in mesenchymal stem cell-mediated immunosuppression in mammalian species. Cell Death and Differentiation.

[171] García-Ortiz A, Serrador JM (2018). Nitric oxide signaling in T cell-mediated immunity. Trends in Molecular Medicine.

[172] Ma OK, Chan KH (2016). Immunomodulation by mesenchymal stem cells: Interplay between mesenchymal stem cells and regulatory lymphocytes. World J Stem Cells.

[173] Robinson DS (2010). The role of the T cell in asthma. The Journal of Allergy and Clinical Immunology.

[174] Ghannam S, Pène J, Moquet-Torcy G, Torcy-Moquet G, Jorgensen C, Yssel H (2010). Mesenchymal stem cells inhibit human Th17 cell differentiation and function and induce a T regulatory cell phenotype. Journal of Immunology.

[175] Cho KS, Park MK, Kang SA, Park HY, Hong SL, Park HK, Yu HS, Roh HJ (2014). Adipose-derived stem cells ameliorate allergic airway inflammation by inducing regulatory T cells in a mouse model of asthma. Mediators of Inflammation.

[176] Park HK, Cho KS, Park HY, Shin DH, Kim YK, Jung JS, Park SK, Roh HJ (2010). Adipose-derived stromal cells inhibit allergic airway inflammation in mice. Stem Cells and Development.

[177] Braza F, Dirou S, Forest V, Sauzeau V, Hassoun D, Chesné J, Cheminant-Muller MA, Sagan C, Magnan A, Lemarchand P (2016). Mesenchymal stem cells induce suppressive macrophages through phagocytosis in a mouse model of asthma. Stem Cells.

[178] de Castro LL, Xisto DG, Kitoko JZ, Cruz FF, Olsen PC, Redondo PAG, Ferreira TPT, Weiss DJ, Martins MA, Morales MM, Rocco PRM (2017). Human adipose tissue mesenchymal stromal cells and their extracellular vesicles act differentially on lung mechanics and inflammation in experimental allergic asthma. Stem Cell Research & Therapy.

[179] Duong KM, Arikkatt J, Ullah MA, Lynch JP, Zhang V, Atkinson K, Sly PD, Phipps S (2015). Immunomodulation of airway epithelium cell activation by mesenchymal stromal cells ameliorates house dust mite-induced airway inflammation in mice. American Journal of Respiratory Cell and Molecular Biology.

[180] Dai R, Liu J, Cai S, Zheng C, Zhou X (2017). Delivery of adipose-derived mesenchymal stem cells attenuates airway responsiveness and inflammation in a mouse model of ovalbumin-induced asthma. American Journal of Translational Research.

[181] Hong GH, Kwon HS, Lee KY, Ha EH, Moon KA, Kim SW, Oh W, Kim TB, Moon HB, Cho YS (2017). hMSCs suppress neutrophil-dominant airway inflammation in a murine model of asthma. Experimental & Molecular Medicine.

[182] Abreu SC, Antunes MA, Xisto DG, Cruz FF, Branco VC, Bandeira E, Zola Kitoko J, de Araújo AF, Dellatorre-Texeira L, Olsen PC, Weiss DJ, Diaz BL, Morales MM, Rocco PRM (2017). Bone marrow, adipose, and lung tissue-derived murine Mesenchymal stromal cells release different mediators and differentially affect airway and lung parenchyma in experimental asthma. Stem Cells Translational Medicine.

[183] Bonfield TL, Koloze M, Lennon DP, Zuchowski B, Yang SE, Caplan AI (2010). Human mesenchymal stem cells suppress chronic airway inflammation in the murine ovalbumin asthma model. American Journal of Physiology. Lung Cellular and Molecular Physiology.

[184] Sugita K, Steer CA, Martinez-Gonzalez I, Altunbulakli C, Morita H, Castro-Giner F (2018). Type 2 innate lymphoid cells disrupt bronchial epithelial barrier integrity by targeting tight junctions through IL-13 in asthmatic patients. The Journal of Allergy and Clinical Immunology.

[185] Wawrzyniak P, Wawrzyniak M, Wanke K, Sokolowska M, Bendelja K, Rückert B, Globinska A, Jakiela B, Kast JI, Idzko M, Akdis M, Sanak M, Akdis CA (2017). Regulation of bronchial epithelial barrier integrity by type 2 cytokines and histone deacetylases in asthmatic patients. The Journal of Allergy and Clinical Immunology.

[186] Tan HT, Hagner S, Ruchti F, Radzikowska U, Tan G, Altunbulakli C (2018). Tight junction, mucin, and inflammasome-related molecules are differentially expressed in eosinophilic, mixed, and neutrophilic experimental asthma in mice. Allergy.

[187] Mathias LJ, Khong SM, Spyroglou L, Payne NL, Siatskas C, Thorburn AN (2013). Alveolar macrophages are critical for the inhibition of allergic asthma by mesenchymal stromal cells. Journal of Immunology.

[188] Takeda K, Webb TL, Ning F, Shiraishi Y, Regan DP, Chow L, Smith MJ, Ashino S, Guth AM, Hopkins S, Gelfand EW, Dow S (2018). Mesenchymal stem cells recruit CCR2. Journal of Immunology.

[189] Ou-Yang HF, Huang Y, Hu XB, Wu CG (2011). Suppression of allergic airway inflammation in a mouse model of asthma by exogenous mesenchymal stem cells. Experimental Biology and Medicine (Maywood, N.J.).

[190] Lin CL, Hsiao G, Wang CC, Lee YL (2016). Imperatorin exerts antiallergic effects in Th2-mediated allergic asthma via induction of IL-10-producing regulatory T cells by modulating the function of dendritic cells. Pharmacological Research.

[191] Kitoko JZ, de Castro LL, Nascimento AP, Abreu SC, Cruz FF, Arantes AC, Xisto DG, Martins MA, Morales MM, Rocco PRM, Olsen PC (2018). Therapeutic administration of bone marrow-derived mesenchymal stromal cells reduces airway inflammation without up-regulating Tregs in experimental asthma. Clinical and Experimental Allergy.

[192] Eiwegger T, Akdis CA (2011). IL-33 links tissue cells, dendritic cells and Th2 cell development in a mouse model of asthma. European Journal of Immunology.

[193] Hammad H, Lambrecht BN (2008). Dendritic cells and epithelial cells: Linking innate and adaptive immunity in asthma. Nature Reviews. Immunology.

[194] Chen L, Zhang W, Yue H, Han Q, Chen B, Shi M, Li J, Li B, You S, Shi Y, Zhao RC (2007). Effects of human mesenchymal stem cells on the differentiation of dendritic cells from CD34+ cells. Stem Cells and Development.

[195] Chen YQ, Shi HZ (2006). CD28/CTLA-4--CD80/CD86 and ICOS--B7RP-1 costimulatory pathway in bronchial asthma. Allergy.

[196] Zeng SL, Wang LH, Li P, Wang W, Yang J (2015). Mesenchymal stem cells abrogate experimental asthma by altering dendritic cell function. Molecular Medicine Reports.

[197] Eljaszewicz A, Wiese M, Helmin-Basa A, Jankowski M, Gackowska L, Kubiszewska I, Kaszewski W, Michalkiewicz J, Zegarski W (2013). Collaborating with the enemy: Function of macrophages in the development of neoplastic disease. Mediators of Inflammation.

[198] Idzkowska E, Eljaszewicz A, Miklasz P, Musial WJ, Tycinska AM, Moniuszko M (2015). The role of different monocyte subsets in the pathogenesis of atherosclerosis and acute coronary syndromes. Scandinavian Journal of Immunology.

[199] Vergadi E, Ieronymaki E, Lyroni K, Vaporidi K, Tsatsanis C (2017). Akt signaling pathway in macrophage activation and M1/M2 polarization. Journal of Immunology.

[200] Eljaszewicz A, Kleina K, Grubczak K, Radzikowska U, Zembko P, Kaczmarczyk P, Tynecka M, Dworzanczyk K, Naumnik B, Moniuszko M (2018). Elevated numbers of circulating very small embryonic-like stem cells (VSELs) and intermediate CD14++CD16+ monocytes in IgA nephropathy. Stem Cell Reviews and Reports.

[201] Cho DI, Kim MR, Jeong HY, Jeong HC, Jeong MH, Yoon SH, Kim YS, Ahn Y (2014). Mesenchymal stem cells reciprocally regulate the M1/M2 balance in mouse bone marrow-derived macrophages. Experimental & Molecular Medicine.

[202] Horwood NJ (2016). Macrophage polarization and bone formation: A review. Clinical Reviews in Allergy and Immunology.

[203] Wang N, Liang H, Zen K (2014). Molecular mechanisms that influence the macrophage m1-m2 polarization balance. Frontiers in Immunology.

[204] Song X, Xie S, Lu K, Wang C (2015). Mesenchymal stem cells alleviate experimental asthma by inducing polarization of alveolar macrophages. Inflammation.

[205] Lambrecht BN, Hammad H (2012). The airway epithelium in asthma. Nature Medicine.

[206] Hammad H, Lambrecht BN (2015). Barrier epithelial cells and the control of type 2 immunity. Immunity.

[207] Wang M, Tan G, Eljaszewicz A, Meng Y, Wawrzyniak P, Acharya S, Altunbulakli C, Westermann P, Dreher A, Yan L, Wang C, Akdis M, Zhang L, Nadeau KC, Akdis CA (2019). Laundry detergents and detergent residue after rinsing directly disrupt tight junction barrier integrity in human bronchial epithelial cells. The Journal of Allergy and Clinical Immunology.

[208] Xiao C, Puddicombe SM, Field S, Haywood J, Broughton-Head V, Puxeddu I (2011). Defective epithelial barrier function in asthma. The Journal of Allergy and Clinical Immunology.

[209] Guida G, Riccio AM (2019). Immune induction of airway remodeling. Seminars in Immunology.

[210] Qin XJ, Zhang GS, Zhang X, Qiu ZW, Wang PL, Li YW, Li W, Xie QM, Ke YH, Lee JJ, Shen HH (2012). Protein tyrosine phosphatase SHP2 regulates TGF-β1 production in airway epithelia and asthmatic airway remodeling in mice. Allergy.

[211] Ge X, Bai C, Yang J, Lou G, Li Q, Chen R (2013). Effect of mesenchymal stem cells on inhibiting airway remodeling and airway inflammation in chronic asthma. Journal of Cellular Biochemistry.

[212] Ogulur I, Gurhan G, Aksoy A, Duruksu G, Inci C, Filinte D, Kombak FE, Karaoz E, Akkoc T (2014). Suppressive effect of compact bone-derived mesenchymal stem cells on chronic airway remodeling in murine model of asthma. International Immunopharmacology.

[213] Urbanek K, De Angelis A, Spaziano G, Piegari E, Matteis M, Cappetta D (2016). Intratracheal Administration of Mesenchymal Stem Cells Modulates Tachykinin System, suppresses airway remodeling and reduces airway Hyperresponsiveness in an animal model. PLoS One.

[214] Firinci F, Karaman M, Baran Y, Bagriyanik A, Ayyildiz ZA, Kiray M, Kozanoglu I, Yilmaz O, Uzuner N, Karaman O (2011). Mesenchymal stem cells ameliorate the histopathological changes in a murine model of chronic asthma. International Immunopharmacology.

[215] Araujo BB, Dolhnikoff M, Silva LF, Elliot J, Lindeman JH, Ferreira DS (2008). Extracellular matrix components and regulators in the airway smooth muscle in asthma. The European Respiratory Journal.

[216] Januskevicius A, Vaitkiene S, Gosens R, Janulaityte I, Hoppenot D, Sakalauskas R, Malakauskas K (2016). Eosinophils enhance WNT-5a and TGF-β1 genes expression in airway smooth muscle cells and promote their proliferation by increased extracellular matrix proteins production in asthma. BMC Pulmonary Medicine.

[217] Kobayashi T, Kim H, Liu X, Sugiura H, Kohyama T, Fang Q, Wen FQ, Abe S, Wang X, Atkinson JJ, Shipley JM, Senior RM, Rennard SI (2014). Matrix metalloproteinase-9 activates TGF-β and stimulates fibroblast contraction of collagen gels. American Journal of Physiology. Lung Cellular and Molecular Physiology.

[218] Hough KP, Curtiss ML, Blain TJ, Liu RM, Trevor J, Deshane JS, Thannickal VJ (2020). Airway remodeling in asthma. Front Med (Lausanne).

[219] Allogeneic Human Cells (hMSC) Via Intravenous Delivery in Patients With Mild Asthma (ASTEC) (2017). https://clinicaltrials.gov/ct2/show/NCT03137199?term=msc&cond=Asthma&draw=2&rank=1. Accessed December 9, 2020.

